# Report from the BIT’s 1st Annual World Congress of Biomedical Engineering Held in Xi’an, China, 9–11 November 2017

**DOI:** 10.3390/medicines4040083

**Published:** 2017-11-16

**Authors:** Gerhard Litscher, Xiaodan Mei

**Affiliations:** 1 Head of the TCM (Traditional Chinese Medicine) Research Center Graz, of the Research Unit of Biomedical Engineering in Anesthesia and Intensive Care Medicine, and of the Research Unit for Complementary and Integrative Laser Medicine, Medical University of Graz, 8036 Graz, Austria; 2 Executive Chair of WCBME 2017, President, BIT Congress Inc., Dalian 116025, China

## 1. Preface

**Figure 1:**
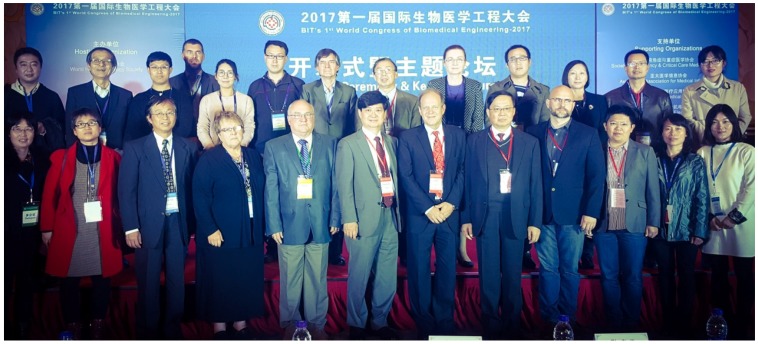
Keynote Forum of the “BIT’s 1st World Congress of Biomedical Engineering 2017”.

**Figure 2:**
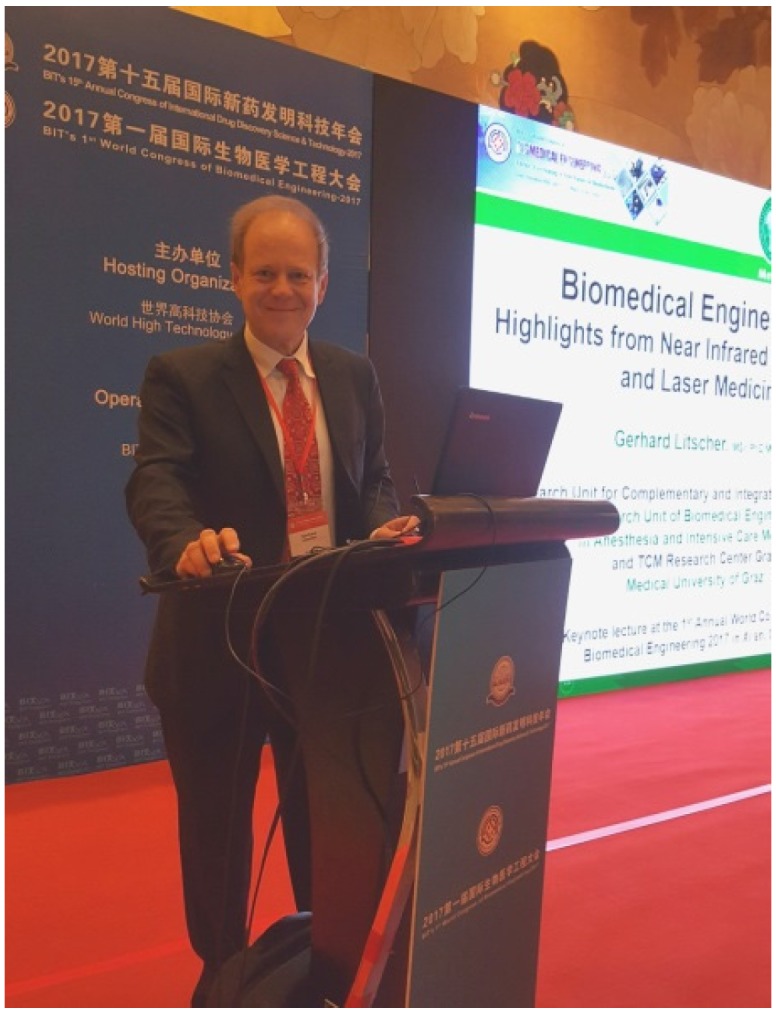
Professor Gerhard Litscher, Keynote Speaker of the “BIT’s 1st World Congress of Biomedical Engineering 2017” in Xi’an.

**Figure 3:**
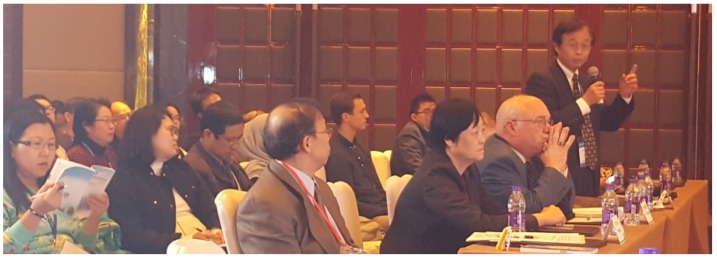
BIT’s 1st World Congress of Biomedical Engineering 2017.

We are delighted to present within this meeting report the abstracts of the “BIT’s 1st World Congress of Biomedical Engineering 2017” which has been hold in Xi’an in China (Figure 1, Figure 2 and Figure 3).

We were very glad that the congress could take place in the exciting, traditional city Xi’an in China. Xi’an is the capital of Shaanxi Province. It is one of the oldest cities in China, and Xi’an is the oldest of the four great ancient capitals, having held the position under several of the most important dynasties in Chinese history. Xi’an is the starting point of the Silk Road and home to the Terracotta Army of Emperor Qin.

This report presents abstracts from an impressive international speaker panel. In total, more than 50 speakers from countries from all over the world like Australia, Austria, Brazil, Canada, China and regions of China, Colombia, Czech Republic, Denmark, France, Georgia, Germany, Hungary, Iceland, India, Italy, Japan, Mexico, New Zealand, Peru, Poland, Qatar, Romania, Russia, Slovakia, Korea, Spain, Switzerland, Republic of Kazakhstan, Turkey, and the United States have presented their latest research results. 

The international term “biomedical engineering” is understood to mean the interaction between medicine and technology for the development of equipment for diagnosis, therapy, and prevention of diseases. Physicians and engineers are equally involved in solving these problems. However, solutions are only possible if the pathological situation or the interaction of all relevant factors is known, so that starting points are identified at which the engineer is able to aid medicine.

Technology has already entered many areas of medicine, as a comprehensive scientific literature review confirms. Just think of X-ray and irradiation devices, measuring and monitoring instruments of various kinds as well as surgery and prosthesis technology, and many others. However, these are not the only research areas addressed in the present congress. In addition, areas are selected that are difficult to access in a technical solution. An attempt is made to interpret the language of the physician in such a way that the technical and scientific core of the problem becomes apparent to the engineer. In the majority of the lectures, therefore, the medical problem is presented, after which the technical aspects of the equipment or research are discussed.

For a better overview, the entire area of biomedical engineering was divided into the following sessions:

- Keynote Forum
- Neural Engineering
- Biomedical Imaging and Biosensors
- Interventional Technology and Treatment
- Digital Medicine and Big Data
- Tissue Repair and Regenerative Medicine
- Cell Engineering
- Nanomedicine and Engineering
- Physical Therapy and Rehabilitation

As editor-in-chief of *Medicines*, I also want to inform you that we have started a Special Issue named ‘New Innovations in Biomedical Engineering’ and that all the readers of this report are cordially invited to submit original papers or short reports especially regarding the lectures of this congress for publication in this Special Issue.

## 2. Keynote Lectures

### 2.1. Geometric Calibration on a Preclinical Cone-Beam Computed Tomography

Chen, J.-H.

**Abstract**

Cone-beam computed tomography (CBCT) systems have been developed to provide anatomic images of patients in order to enhance the accuracy of diagnosis. In the reconstruction process, accurate geometry is extremely critical to obtain high quality reconstructed tomographic slices. Especially in high resolution CBCT, a bit of misalignment in geometry can seriously degrade the quality of the reconstructed images. There are several geometric calibration methods developed which use a dedicated phantom to acquire geometric parameters. The projections with geometric misalignment can be calibrated by re-mapping the projections with the estimated geometric parameters. Then, the observed object can be reconstructed with the calibrated projections. In this study, a new phantom which consists of 26 steel balls precisely located in a cylindrical-shaped structure is applied to calibrate the geometry of a preclinical CBCT system. There are two circular patterns in the cylindrical phantom which consists of 12 steel balls spaced evenly over 360 degrees in each circle. The remaining two steel balls are located at the center of the two sides of the cylindrical phantom. The calibration process with the proposed phantom is described in the following. First, the projections of the two circular steel ball patterns were utilized to fit two ellipses by least squares. Second, the converging points of the two ellipses were calculated by the elliptical functions. Third, the magnifying factor is estimated by the other two steel balls. Finally, the complete geometric parameters including three rotation parameters of the detector, the location of the piercing point and the source point position can be obtained by solving the analytical equations. After that, the geometric parameters are applied to re-mapping the projection data. In order to validate the proposed method, a prototype preclinical CBCT gantry is applied. The proposed calibration phantom and the hydroxyl-apatite (HA) phantom were scanned by the real gantry system. After geometric calibration by re-mapping the projection data with the geometric parameters, the HA phantom images were reconstructed by the filtered back projection (FBP) algorithm with the calibrated projections. Comparing before and after calibrated reconstructed slices of the HA phantom, the artifacts caused by misalignment of geometry are obviously reduced.

**Biography**

Dr. Jyh-Cheng Chen received the B.S. degree in physics from National Central University, Taiwan, in 1983, and the M.S. degree in physics and Ph.D. degree in optical sciences from the University of Arizona, USA in 1988 and 1995, respectively. In 1995, he joined the Opto-Electronics and System Laboratories, Industrial Technology Research Institute, Taiwan, as a Research Associate working on semiconductor laser packaging. In 1996, he became a member of the faculty of Division of Radiological Science and Technology, Department of Medical Technology, National Yang-Ming University (NYMU), Taiwan. In 1998, he became an associate professor of Institute of Radiological Sciences, NYMU, Taiwan. Since 2005, he has been a Professor in the Department of Biomedical Imaging and Radiological Sciences (BIRS), NYMU, Taiwan.

### 2.2. The Development and Implementation of Stroke Risk Prediction Model in National Health Insurance Service’s Personal Health Record

Cho, K.

**Abstract**

In Korea, PHR is being used to make a nationwide approach to improve the health of each individual.

Cancer, cerebrovascular disease, diabetes, etc., and provides a method of disease prevention for each individual. In the case of stroke, a 10-year stroke prediction model was developed and categorized as a stroke using the Korean national health examination data. Then, we will develop the algorithm to provide a personalized warning on the basis of each level of stroke risk and a lifestyle correction message about the stroke risk factors.

The AUC values of stroke risk prediction models, developed using multiple risk factors, were 0.83 in men and 0.82 in women, which showed a high predictive power. The probability of stroke within 10 years for men in the normal group (less than 50 percent) was less than 3.92 percent and in the very risky group (top 0.01 percent) was 66.2 percent and over. The women’s probability of stroke within 10 years was less than 3.77 percent in the normal group (less than 50 percent) and 55.24 percent and over in the very risky group.

A lifestyle correction message by each risk factor was developed and set up automatically in the individual PHR.

**Biography**

Professor Cho is currently the President of APAMI (Asia-Pacific Association of Medical Informatics), Chair of Health informatics committee, NHIMC Ilsan hospital, Professor of Yonsei medical colleges. He majored in M.D and Family Medicine at Yonsei University College of Medicine, Master of health policy at the Graduate School of Public Health, Ph.D. at the graduate school, and Geriatric fellowship at the University of Rochester, USA for two years. Many projects have been carried out in the field of family medicine, geriatrics, health policy, quality improvement, and medical informatics, with over 40 of papers and book publications. Since 2009, he has been in charge of the national health information exchange project DCM project PI, for three years from 2012, the head of NHIMC Hospital EMR, from 2014 onwards, he is currently in charge of the National Disease Prediction Project. Currently, he is a member of National Medical Information Standardization Committee and DCM committee chair.

### 2.3. Biomedical Engineering—Highlights from near Infrared Spectroscopy and Laser Medicine

Litscher, G.

**Abstract**

The application of near-infrared spectroscopy (NIRS) in different fields of research has gained importance, and the number of scientific studies has grown extensively. Within the current keynote lecture, a retrospect and highlights from 1995 to 2017 from the interdisciplinary NIRS research team at the Medical University of Graz, Austria, Europe will be presented. The main topics are results from transcranial cerebral oximetry in the hyperbaric environment and the non-invasive assessment of oxygen metabolism using NIRS technology during high altitude trekking in the Nepal Himalayas. In addition, NIRS publications from intensive care medicine and laser acupuncture are shown. Last but not least, the application of regional oxygen saturation in regenerative medicine is demonstrated. NIRS could be a fruitful approach helping to explore deeper biological mysteries within the brain and the periphery. Special results from laser medicine will also be presented briefly.

**Biography**

Prof. Gerhard Litscher is a biomedical engineer and the head of the Research Unit for Complementary and Integrative Laser Medicine, of the Research Unit of Biomedical Engineering in Anesthesia and Intensive Care Medicine, and of the TCM Research Center Graz at the Medical University of Graz, Doctor of technical and Doctor of medical sciences, international lectures, about 600 scientific publications (>190 of them SCI/PubMed-listed), partly on biomedical engineering and on basic acupuncture research, author and/or editor of 13 books, currently editor-in-chief and/or member of the editorial board of more than 35 international scientific journals (e.g., Editor-in-chief of the Internet Journal of Alternative Medicine (IJAM), Medicines, Integrative Medicine International, Integrative and Complementary Medicine; Associate Editor for Medical Acupuncture, Associate Editor of the Journal of Acupuncture and Meridian Studies (JAMS), one of the editors and lead guest editor of Evidence-based Complementary and Alternative Medicine (eCAM), and Associate Editor of BMC Complementary and Alternative Medicine). Prof. Litscher’s special interests are computer-based High-Tech Acupuncture Research and Neuromonitoring. He is University Professor at the Medical University of Graz and Honorary/Guest Professor at eleven institutions and universities in Asia.

## 3. Lectures

### 3.1. Neural Engineering

#### 3.1.1. Remote Biomedical Engineering via Mastering the Geometrical Optics of Chromosomes

Caesar, I.

**Abstract**

The same gene is expressed in functional individuals and species by metacentric chromosome, and in dysfunctional individuals and species via acrocentric chromosome. Metacentric chromosome is analogous to a well-centered and focused eye crystal, while acrocentric chromosome is analogous to a myopic or farsighted eye. Chromosome is a lens that allows our cells to focus the needed wave information during cell division, analogously to a crystal in our eye. Thus, Biomedical Engineering is possible only via mastering the Geometrical Optics of Chromosomes by Laser Bioholography. Current genetic engineering via cutting and pasting DNA snippets destroys the systematic wholes of chromosomal bioholograms and the refraction of the geometrical optics in chromosomes, and, therefore, causes genetic collapse. The Quantum Leap in the Biomedical Engineering is the cost of our survival. Biomedical Engineering as Bioholography is based on Quantum Physics, and understanding the non-local nature of the Bioholograms. Since universe is holographic, we can record and transmit only holographic information, based upon changing the refraction of the scalar wave diffraction grating in chromosomes. According to Holographic principle, Universe is entirely in its every matrix point. The implication of the Holographic Principle states that if Universe is entirely in its every matrix point, then, every matrix point is not simply different from any other matrix point, but is unique. This means that every wave matrix is both non-local, and unique. Thus, if we have two exact copies of the same non-local wave matrix, we can transmit information instantaneously and remotely, from one copy of the same non-local unique wave matrix to another copy. Thus, the precision in the Biomedical Engineering is achieved not via the precision of localization, but, vice versa, via the precision of refraction in the unique and non-local bioholograms aka “wave crystals”. The secret in the remote Biomedical Engineering as Quantum Biocomputing lies in the recording and instantaneous transmission of a structural phantom. I will introduce the new method for recording and remote transmitting of bioholograms via laser spectroscopy and coding electrets on nanolevel via laser. The technology creates “lenses” assisting our chromosomes in correcting their refraction for focusing genetic bioholographic information, both locally and remotely.

**Biography**

Irene Caesar, Ph.D., is a Founder of Matrix City LLC, Russia, company creating global subscription platforms for bioelectronic drugs (founded 2016); Founder and President of Wave Genome LLC, company producing bioelectronic devices and software for the distribution of bioelectronic drugs for rejuvenation only (founded 2010); Co-founder of “Matrix City” Consortium with the Institute for National Security in Moscow for building self-sufficient human settlements based upon the remote management of biosystems, climate and geophysical processes for the first time in the history of humankind (founded 2012), presented in the Honorary Lecture at the Harriman Institute of the Columbia University in September 2012; Co-founder of the Quantum Biointernet for the remote rejuvenation via distant laser signal, commercially offered for the first time in the history of humankind by Irene Caesar’s company Wave Genome in May 2013; Colonel of Irkutsk Cossack Military, awarded the Medal of Faith and Service to Russia (2014). Dr. Caesar received her doctoral degree from the Graduate Center of the City University in New York.

#### 3.1.2. A Potential Activation Blocking Mechanism of High Frequency Stimulation on Axons

Mei, L.

**Abstract**

High-frequency stimulation of dorsal column axons and their potential mechanisms for achieving pain relief was explored. A threshold charge rate curve was developed to locate a critical axonal node in an axon to determine where and when action potentials (APs) may be generated. Blocking of the axon’s ability to generate APs occurred if an equivalent current generated by the field potential gradient i_ext_ was negative, related to being at the first node of the activating function virtual anode and if its absolute value was larger than that generated by the transmembrane potential gradient, i_axo_. In high-frequency stimulation (>4–5 kHz), the M-gate at the first node of the virtual anode is locked in the closed position, while the H- and N-gates are locked open. Therefore, the ion current i_ion_ is small and can be ignored. This blocking phenomenon only occurs in large diameter fibers in response to a monophasic stimulus.

**Biography**

Mr. Longzhi Mei is senior research assistant of Department Neurosurgery, Beth Israel Deaconess Medical Center, Boston, MA, USA. He is interested in Neural Simulation and has published 6 papers in electrode stimulation and mechanisms of action in neuromodulation devices. He received his B.S. in Physics at Chengdu University of Science and Technology, China in 1982, and taught general physics in the Southwest Agricultural University 12 years. He then went studied in the United States, receiving an M.A. in Biology at Lehman College, City University of New York, USA 1996. He received his M.S. in Computer Science, Rivier College, New Hampshire, USA.

#### 3.1.3. Modelling Cognitive Laws for Recognizing Emotions

Wen, G.

**Abstract**

Among signals such as psychological signals and forehead biosignals, facial expression is the most effective way for humans to express emotions. This is why it has attracted much attention in machine learning applications in recent years. Typical applications include emotional health, sentiment analysis, and human-computer interaction. Generally, facial expression recognition is performed by machine learning methods. However, their performance are far away from that of human being, as they did not consider significant cognitive laws. For example, for human being, the inertial thinking schemes can be formed through learning, which are then applied to quickly solve the coming similar problems. When problems are heavily different, the inertial thinking generally present the solutions that are definitely imperfect. In such case, Human being will apply the creative thinking such as the reverse thinking to solve problems. Although machine learning methods also form the inertial thinking through learning the rules from a large amount of data. However, when the testing data are of much difference, the formed inertial thinking will inevitably generate errors. This inertial thinking is called as the illusion inertial thinking. Because all machine learning methods do not consider the illusion inertial thinking, it is suggested that the reverse thinking can be considered. It increases the generalization ability of the machine learning methods. The conducted experimental results on benchmark datasets validated the proposed method.

**Biography**

Guihua Wen is a professor of Artificial Intelligence (AI) and head of machine learning and data mining group in South China University of Technology, Guangzhou, China. He majors in emotion recognition, deep learning, large scale data mining, and applications to health care, including Traditional Chinese Medicine. Currently his research are funded by 4 projects from national and local governments. He has published over 30 papers in the international conferences such as famous AAAI and IJCAI, and journals such as Pattern Recognition and Appl. Soft Comput. His research achievement on facial expression recognition was ever reported by Yangcheng Evening News in China.

### 3.2. Biomedical Imaging and Biosensor

#### 3.2.1. Understanding the Cardiac Varices with the Aid of Technology

Segura-Saldana, P.

**Abstract**

Cardiac varices are a very little described entity, many consider it as rare; But our work group considers that it is underdiagnosed, because it is usually asymptomatic, and with routine imaging methods like Chest X-ray or echocardiography are usually imperceptible, this is when Cardiac Magnetic Resonance becomes important, but it is still expensive and an examination usually takes 2 to 3 h, reasons why cardiac magnetic resonance is not done routinely. We have developed an abbreviated protocol in Magnetic Resonance to do these exams in approximately 20 min. In addition, we are working on SDR (SOFTWARE DEFINED RADIO) and algorithms of correction by mistake, we believe that we can optimize the obtained in the conventional magnetic cardiac resonance and with this to improve the diagnosis of cardiac varices.

My presentation will be about the problem of diagnosing an entity that is rare, to keep it in mind. And also the treatment and prognosis of this kind of patients.

**Biography**

Pedro Segura-Saldana, I am cardiologist at the Rebagliati Martins Hospital (the biggest hospital of my country), CEO of a Biomedical Company in Peru, research and work focused on solving cardiologic, metabolic and oncological problems from a technological perspective. I am a part-time Professor of Biomedical Engineering in the Dual Degree Program of the Universidad Peruana Cayetano Heredia and of the Pontifical Catholic University of Peru. I have published several articles in journals in my country and also in scientific journals at the United States of North America and Europe, I won Grants for the development of medical devices like arrhythmia detector and the infarct in real time with cellular technology, monitoring the Central Blood Pressure in a non-invasive way, also shortened Magnetic Cardiac Resonance protocols (Grant funded by the UK) among others and I am currently developing intelligent methods in the processing of cardiac magnetic resonance. I am a current member of the Peruvian Society of Cardiology and the Peruvian Society of Hypertension. We are carrying out the National Registry of Cardiac Tumors in Peru, will be the first National registry of cardiac tumors.

#### 3.2.2. To Map the Local Temperature Distribution of a Single Live Cell

Xu, S.

**Abstract**

To monitor the temperature distribution of a cell and its changes under varied conditions is an important fundamental issue and currently remains as a technical challenge. A variety of non-contact methods used for measuring cellular temperature have been developed, where changes of local temperatures at cell-level and sub-cell-level are indirectly calculated through the changes in intensity, band-shape, bandwidth, lifetime or polarization anisotropy of the fluorescence spectra recorded from the nano-sized fluorescent materials pre-injected into the target cell. Unfortunately, the optical properties of the fluorescent nano-materials may be affected by complicated intracellular environment, leading to unexpected measurement errors and controversial arguments. We have developed high-performance Pd/Cr micron thin-film thermocouple (TFTC) arrays and double-stabilized system with a stability of ±5 mK. The local temperature of TFTCs close to adherent human hepatoblastoma (HepG2) cellswere continuously recorded for days, showing frequent temperature increment of 10–60 mK and a maximum up to 200–300 mK. Details of the device fabrication, sample preparation, thermal system stabilization and measurement procedures are published elsewhere. The method has set up solid foundation for realization of real-time and precise 2D mapping of local temperature distribution for a single live cell with our nano-sized TFTC arrays, therefore it may valuable information for cell biology and research on drug effects and clinical therapies.

**Biography**

ProfessorXuobtained his Ph.D. degree in 1999 from Department of Physics, National University of Singapore. He worked at the “Center for Superconducting & Magnetic Materials”, National University of Singapore as a Research Fellow in 1999–2001, at Physics Department, Pennsylvania State University as a postdoctoral researcher 2001–2003, and then at “Center for Nanoscale Science”, Pennsylvania State University as a Research Associate in 2003–2006. Since April 2006 he has been teaching and doing research at the Department of Electronics, Peking University. Currently Prof. Xu’s group mainly works on the underlying physics of electrical communication in biosystems, including soft-material electromagnetic (EM) waveguides, propagation of EM pulses in axons and among cells, working mechanism of brain, etc. The group also works on time-resolved 2D mapping of temperature at the micro-/nano-scales, e.g., a single cell, 3D micro-bio-probes, and solid thermal sensors for ultrahigh temperatures (>2000 K).

#### 3.2.3. Rigid Endoscopic Laser Diverticulotomy for Lateral Pharyngoesophageal Diverticuli, New Concept and Method

Lee, C.-J.

**Abstract**

Killian-Jamieson diverticulum (KJD) is a rare cervical esophageal abnormality. Transcervical approach has been the main treatment modality to prevent recurrent laryngeal nerve (RLN) injury. We presented several cases of patients confirmed with KJD and were managed successfully under rigid endoscope. The new technique and idea were described in detail. Under rigid laryngoscope, the septum between the true esophagus lumen and diverticulum can be exposed clearly. A microscope equipped with CO_2_ LASER system offered precise and focused point cutting energy to the septum. Several efforts were applied to prevent RLN injury in the cases descriptions. We use transnasal esophagoscope and eating assessment tool (EAT-10) for anatomic and functional result evaluation. Much improved symptoms of dysphagia and intact RLN function were observed. Under the assist of rigid laryngoscope and point-cutting CO_2_ LASER, KJD diverticulotomy could be performed safely with little complication for patients refusing transcervical route.

**Biography**

Dr. Chia-Jung Lee received an M.D. degree from China Medical University, school of Medicine in Taichung, Taiwan, and completed his clinical training in Shin-Kong Wu-Ho-Su Memorial Hospital in Taiwan and with advanced training in Laryngological Surgery at the University of Washington in Seattle with Professor Albert Merati on 2012. He then came back to Taiwan and establish the lab of voice and swallowing in Shin-Kong Wu-Ho-Su Memorial Hospital on 2013. Dr. Chi-Jung Lee focused his clinical interests at airway disease, dysphagia disease and phonosurgery and helps a lot of patients with laryngeal diseases in Taiwan and Southeast Asia. He was the invited speaker of the 13th Asia-Oceania Otolaryngology Head and Neck Congress on 2015. Dr. Chia-Jung Lee holds a faculty appointment in the Shin-Kong Wu-Ho-Su Memorial Hospital, Department of Otolaryngology-Head and Neck Surgery. He was promoted as the Chief of the Otolaryngologic Endoscopic room since 2014.

#### 3.2.4. Preparation and Integration of Multichannel Microfluidic Photonic Biosensors

Heer, R.

**Abstract**

Inkjet printing is a versatile method to apply surface modification procedures in a spatially controlled, cost-effective and mass-fabrication compatible manner. Utilizing this technology, we investigate two different approaches for functionalizing label-free optical waveguide based biosensors: (a) surface modification with amine-based functional polymers (biotin-modified polyethylenimine (PEI-B)) employing active ester chemistry and (b) modification with dextran based hydrogel thin films employing photoactive benzophenonecrosslinker moieties. Whereas the modification with PEI-B ensures high receptor density at the surface, the hydrogel films can serve both as a voluminous matrix binding matrix and as a semipermeable separation layer between the sensor surface and the sample. We use the two surface modification strategies both individually and in combination for binding studies towards the detection of the protein inflammation biomarker, C-reactive protein (CRP). For the specific detection of CRP, we compare two kinds of capture molecules, namely biotinylated antibodies and biotinylated CRP-specific DNA based aptamers. Both kinds of capture molecules were immobilized on the PEI-B by means of streptavidin-biotin affinity binding. As transducer, we use an integrated four-channel silicon nitride (Si_3_N_4_) waveguide based Mach-Zehnderinterferometric (MZI) photonic sensing platform operating at a wavelength of 850 nm (TM-mode).

Keywords: photonic-sensors, integrated-optical-waveguides, inkjet-printing, hydrogel, polymer, surface modification, C-reactive protein.

**Biography**

Dr. Rudolf Heer is leading the nano components research group at the Austrian Institute of Technology—AIT in Vienna, Austria. He is expert for the design and fabrication of nano scale devices for sensors. His major accomplishments are the realization of a wireless online monitoring system for adherent cell cultures in microtiter-plates, the acceleration of incubation processes in DNA bio-chips by magnetic particles and the transfer to a technology agency for commercialization and the realization of a floating electrometer for scanning tunneling microscope applications in the femtoampere range. He graduated in electrical engineering at the Vienna University of Technology in 1996. During his diploma thesis he deepened his knowledge in laser physics. His doctorate study focused on low temperature semiconductor physics. He was engaged as a lecturer as well. After the conferral of doctorate in 2000 he joined EPCOS OHG in Munich and was responsible for the integration of radiofrequency front end modules for cell phones. In 2002 he moved to Applied International Informatics AG in Vienna and focused on sizing and performance tuning of SAP/R3 systems. Since 2004 he is leader of the nano components research group at the AIT. Rudolf Heer authored more than 50 scientific publications.

#### 3.2.5. Tunable Uptake/Release Mechanism of Protein Microgel from Microdroplets Inbiomimicking Environment

Simone, G.

**Abstract**

Microgels are intra-molecular crosslinked macromolecules which can be used as vehicles to deliver drugs into point-of-use inside the patient’s body and release drugs only there minimizing unwanted side effects. The microgels can be influenced by many different stimuli including pH, ionic strength, temperature and mechanical stresses. The gelatin microgels were formed from microdroplets in a microfluidic channel and stabilized in-situ by aldehydes, thus freezing them into a spheroidal shape. The microgel morphology and its response to external stimuli such as pH and ionic strength of the suspending media were characterized. It was found that the diameter of the spheroidal microgels was sensitive to both, pH as well as ionic strength. In fact, the distribution of the charges into the microgels affected the behavior of swelling and uptake.

In conclusion, the microgel uptake of the molecules such as Rhodamine B and Methylene Blue were investigated, to mimic drug uptake/release mechanisms. Upon physiological conditions, the uptake of Rhodamine was rapid and a uniform distribution the fluorescent molecules was recorded inside the microgels. However, the mechanism of release became faster upon acid conditions, mimicking stomach environment.

Upon physiological conditions, Methylene Blue release resulted faster than Rhodamine.

Dependence of uptake and release of model drugs on basic/acid conditions shows that the microgels could be used for targeted drug delivery. Different shape of the microgels such as sphere, spheroids, and rods could be useful in tissue engineering or during the mechanisms of vascularization.

**Biography**

Dr. Giuseppina Simone obtained her Master in Chemical Engineering from the University of Naples “Federico II” (Italy) in 2001, and her Ph.D. in Mechanical Engineering from University of Rome “La Sapienza” (Italy) in 2007. During her doctorate, she was visiting scientist at the MIC Department of the Technical University of Denmark. Afterwards, she did her research in several laboratories including Silicon Biosystems S.p.A., Harvard Medical School, Kist Europe and most recently at the Italian Institute of Technology. In 2016 she became Associate Professor at the NPU at the Department of Mechanical Engineering. Dr. Simone’s scientific interests include Microfluidics and miniaturization, Lab on a Chip, Microfabrication, Soft Matter and Biophysics. Dr. Simone has published more than fifty of papers, patents and books.

#### 3.2.6. Computer Aided Quantification of Tumor Burden for Assessment of Treatment Response in Cancer: Evidence Based Overview

Singh, A.

**Abstract**

Tumor response evaluation in diagnostic imaging has evolved over the past 40 years. The World Health Organization (WHO) response criterion was first introduced in 1979 without any specific imaging stipulations or protocols. Therefore, different groups subsequently proposed modifications and to unify and standardize the criteria, the Response Evaluation Criteria In Solid Tumors (RECIST) was introduced in 2000. However, practical use of RECIST together with the rapid development of imaging techniques and newer chemotherapeutic agents has highlighted the limitations of RECIST and the need for updated criteria. Various newer anticancer drugs that vary in their effects to induce necrosis in the tumor content have demonstrated an inherent limitation and unsuitability of RECIST-based tumor evaluation that assesses only lesion size by single dimensional measurements. Volumetry by automated techniques estimates total tumor volumes and includes necrosis volumes; a tumor fraction that is actually considered a response to therapy. We believe that assessing the volume change of merely the non-necrotic content of the tumors or dynamic cross-sectional imaging-based viable tumor tissues between the pretreatment and post treatment time point imaging scans would be a more reliable predictor of therapy response than the existing RECIST criteria. The illustrated examples in this presentation with in-house conducted research for such methods provides evidence-based support for automated extraction of necrotic components from the tumor tissue to derive the non-necrotic/viable tumor volume estimates that may provide a valid and reliable option for its incorporation with the present diagnostic workflow in the future. This presentation will educate on the limitations of currently used response assessment criteria and will focus on a new set of preliminary studies that will highlight the emerging importance of computer-aided volumetry in treatment response assessment in oncology.

**Biography**

Following his medical school training, Dr. Anand Singh was selected among the top ten physicians to pursue my MD program from one the best cancer centers in Asia. He is currently a clinical emergency radiologist and research faculty in the department of Radiology at Massachusetts General Hospital (MGH), Harvard Medical School and member of Dana-Farber/Harvard Cancer Center, USA. His research interests have increasingly inclined on development of newer Computer-aided detection and tumor imaging objective methods over span of thirteen years in Harvard. Dr. Singh’s research career at MGH has resulted in over 140 publications in form of original research articles, peer review articles and book chapters. There are over 600 citations of his research across the globe and he has received several awards, including awarded the Radiological Society of North America (RSNA) research trainee awards, three times (2010 and 2012) and RSNA research scholar grant (2009) at an international platform that is considered to be one of the largest medical conferences in the world. His research work has been instrumental in getting grants nearly 2 Million $ for his research. His research work has also led to formation of computer design and up gradation projects in his lab where automatic quantification of viable liver and sarcoma tumor volumes are under advanced development for clinical translation with promising results. Dr. Singh’s work has also led to innovations of multiple clinical imaging protocols for delineation of liver, pancreatic, biliary, and renal vasculature and bile duct anatomy. Dr. Singh has served as a lead moderator of IAEA (International Atomic Energy Association) weeklong education course for CT radiation dose with participants as representatives from 11 IAEA affiliated countries from Europe and Asia. Dr. Singh is actively involved in MGH and Harvard Global health and outreach wherein his efforts span across various continents in collaborations.

#### 3.2.7. Correlated Images of the Biological Samples by Using Far Field and near Field Microscopy Techniques

Stanciu, G.A.

**Abstract**

In our work we present some investigations on different tissues acquired by using several linear and nonlinear optical microscopy techniques working in far field or in near field. For imaging we used a new multimodal system integrating several microscopy techniques which offer the possibility for investigations at micro and nanoscale on the same area. Far field techniques including confocal laser scanning microscopy (CSLM) and second harmonic generation imaging (SHGI) have hundred nanometers resolution and scattering scanning near field optical microscopy(s-SNOM) has a nanometer resolution. Optical images are correlated with surface topography images acquired by atomic force microscopy (AFM). Our work is mainly focused on collagen structures.

**Biography**

Dr. George A. Stanciu, is Professor of Physics at University Politehnica of Bucharest. He is head of Center for Microscopy-Microanalysis and Informatiion Procesing founded by him in 2001. He got his Doctor degree (Ph.D.) in Technical Physics in 1981. He received the Professor title in 1994. Starting with 1973 he has been working in the Laser Scanning Microscopy field. In 1977 his team reported the first Digital Laser Scanning Microscope. He have been working for long time in the frame of laser scanning microscopy (instrumentation and applications) are and also in the scanning probe microscopy (instrumentation and applications). Lately he is focused on biological application of different scanning laser microscopy techniques. Prof. George Stanciu is senior member of the IEEE from 1995.

#### 3.2.8. High-Sensitivity and Broadband Graphene-Based Detector for in Vivo Photoacoustic Microscopy

Song, W.

**Abstract**

Photoacoustic microscopy (PAM) is capable of measuring the optical absorption properties in the tissues, which is complementary to the sophisticated optical microscopic technologies, such as confocal microscopy, multiphoton microscopy, and optical coherence tomography, based on various imaging contrasts, including optical scattering, fluorescence, and polarization. The optical diffraction-limited lateral resolution down to the micrometer or even submicrometer scale is achieved by utilizing a microscope objective to tightly focus the photoacoustic excitation laser onto biological samples. However, both acoustic detection sensitivity and frequency response is limited by the piezoelectric ultrasonic transducer, which produces low signal-to-noise ratio and poor axial resolution in PAM. Here, we report a novel ultrasonic/photoacoustic detection method based on sandwiched configuration of graphene between prism and water. Photoacoustic initial pressure transients modulate refractive index of the coupling water, which changes the polarization-dependence absorption of the graphene. As a result, the phtoacoustic detection is realized through recording the reflectance perturbation of the probing beam with a balanced photodiode. The graphene-based sensor shows an estimated noise-equivalent-pressure sensitivity of around 550 Pa over an approximately linear pressure response from 11 kPa to 55 kPa. Moreover, it enables much broader ultrasonic bandwidth detection up to about 150 MHz, primarily associated with the highly localized evanescent field. In vivo PAM imaging of three-dimensional microvasculatures is obtained label-freely in mouse ears because of strong optical absorption of inherent hemoglobin. Our results implies the great potentials of graphene-based PAM for biomedical investigation, such as tumor angiogenesis and neurovascular coupling.

**Biography**

Dr. Wei Song is working as Associate Professor in Nanophotonics Research Centre, Shenzhen University. In 2014, he obtained his Ph.D. degree in Department of Physics, Harbin Institute of Technology. Afterwards, he started work as Assistant Professor at Shenzhen Institute of Advanced Technology, Chinese Academy of Sciences. His main research interests are biomedical photonics, including photoacoustic imaging, optical coherence tomography (OCT), multiphoton microscopy, and multimodal microscopic technologies by integrating multiple imaging methods. Dr. Song has published over 20 peer-reviewed papers on the development of novel photoacoustic microscopy, OCT, and multimodal microscopy.

### 3.3. Interventional Technology and Treatment

#### 3.3.1. Using Crosslinked Polyisobutylene, a Novel Medical Biomaterial, to Develop Next Generation of Intraocular Lens for Cataract Treatment

Sun, R. and Pinchuck, L.

**Abstract**

Xi’an Pillar BioScience Co., Ltd. (Xi’an, China) is an ophthalmology company founded in January, 2015. Pillar’s mission is to develop the world’s first pre-loaded, hydrophobic, glistening-free, micro-insertable intraocular lens (“IOL”) for use as both a commodity and premium IOL to serve global cataract patients. Work on this novel next-generation IOL material made from crosslinked polyisobutylene -[CH_2_-C(CH_3_)_2_]_n_- (“xPIB”) began in 2003. Implantable medical devices made from polyisobutylene co-polymers (e.g., SIBS) have been implanted in more than a million patients as drug-delivery coatings for coronary stents since 2004 and have been used in the eye as a glaucoma shunt in over 1000 patients since 2008. XPIB is made ultra-pure with no cleaveable groups; thereby eliminating the possibility of forming glistenings or hazing typical of acrylic-based IOLs. Its high refractive index (1.50–1.53), rubbery nature (Modulus at 10% strain < 100 g/mm^2^), scratch resistance and low glass transition temperature (−70 °C) enables insertion into the eye through cannulas as small as 1.5 mm; thereby eliminating suturing of the cornea. Bonded-in UV blockers provide a UV cut-off at <400 nM with light transmission values of 95%. It’s Abbe number at 23 °C is 49.8. The unique chemistry of xPIB provides distinct advantages over conventional hydrophobic materials (e.g., acrylic) in regard to lack of glistening, low insertion profile, compatibility with silicone oils (vitrectomy patients) and clarity. In addition, it can be injection molded or machined to provide high-quality affordable lenses. From our development, we have demonstrated that the IOLs from xPIB showed significant advantages in both optical and mechanical properties compared to the existing leading IOL products in the markets.

**Biography**

Dr. Roger Sun is the Chairman & CEO for both Xi’an Pillar Bioscience Co. Ltd. and Xi’an Renalysis Medical Technologies Co. Ltd. based in Xi’an, China. Pillar is using xPIB to develop series of therapeutic medical devices including its leading product—intraocular lens. Renalysis is developing peritoneal dialysis products including non-PVC packaging materials for renal failure patients. Dr. Sun has published more than 30 papers in peer-reviewed international journals since he started his career as an ophthalmologist in 1995. He received his doctoral degree with honor from Health Science Center of Beijing University in 1998, then he moved to USA to continue his ophthalmic training as a postdoctoral fellow in Indiana University. Over there, he won series of international recognized awards including the Outstanding Young Ophthalmic Researcher Award, and became a world recognized expert in eye research. From 2006, he focused his career in investment and acquisition of the first class and innovative technologies for medical applications including medical devices and pharmaceuticals for Bayer Healthcare Corporation and Baxter Healthcare Corporation, respectively. In his tenure, he completed more than 5 billion USD acquisition transactions, and more than 500 million USD investment returns with more than 11 times return rate. Dr. Sun received his MD from Health Science Center of Beijing University and his MBA from Kelley School of Business of Indiana University.

Dr. Len Pinchuck became a member of US National Academy of Engineering (NAE) in 2012, and was elected as a member of the Selection Committee of NAE in 2015. Dr. Pinchuck has more than 115 granted invention patents in biomaterial, and published more than 30 papers in peer-reviewed international journals. He is a very well-known world expert in biomaterials for medical applications. With his inventions, more than 100 billion USD medical products have been commercialized globally, including the first expandable endoprosthsis and the first coated coronary stents. Dr. Pinchuck has founded or co-founded series of medical device companies using his own inventions to develop different medical products for hard to treat diseases such as liver cancer and glaucoma. In 1996, his first company, Cortiva Corporation., was acquired by Pfizer, Inc. Most recently, one of his companies, Infocus, Inc., was acquired by Santen Pharmaceuticals at more than 1 billion USD. Dr. Pinchuck received his Ph.D. in chemistry and medicine and M.S. in biomedical engineering from University of Miami, and his BSc in chemistry from McGill University in Canada.

#### 3.3.2. A Simple, Less Invasive Stripper Micropipetter-Based Technique for Day 3 Embryo Biopsy

López-Bayghen, E., Cedillo, L., Ocampo, A. and Camargo, F.

**Abstract**

A key step of for Preimplantation genetic testing (PGT) for in vitro fertilization (IVF) is blastomere removal, that has many technical issues. Here, we compared a more simple procedure based on the StipperMicropipetter, named S-biopsy, to the conventional aspiration method. On Day 3, 368 high-quality embryos (>7 cells on Day 3 with <10% fragmentation) were collected from 38 women. For each patient, embryos were equally separated between the conventional method (*n* = 188) and S-biopsy method (*n* = 180). The conventional method was performed with the standardized protocol. For the S-biopsy method, a laser was used to remove a significantly smaller portion of the zonapellucida. Afterwards, the complete embryo was aspirated with a Stripper Micropipetter, forcing the removal of the blastomere. Selected blastomeres went to PGT using CGH microarrays. The S-biopsy and the conventional method did not differ in embryo integrity (95.0% vs. 95.7%) or blastocyst formation (72.7% vs. 70.7%), aneuploidy rate was similar between the two methods (63.1% vs. 65.2%). However, the time required to perform the S-biopsy method (179.2 ± 17.5 s) was significantly shorter (5-fold) than the conventional method. The S-biopsy method is comparable to the conventional method that is used to remove a blastomere for PGT, but requires less time. Furthermore, due to the simplicity of the S-biopsy technique, this method is more ideal for IVF laboratories.

**Biography**

Doctor Esther López-Bayghen is a Main Researcher in Mexico’s leading research center, CINVESTAV, well as the head adviser of Research and Development for the Ingenes Institute in Mexico City. She has an extensive background investigating mechanisms, impact and possible applications for gene expression data, publishing more than 55 papers in areas as cell biology and tissue engineering. She completed her Ph.D., finalizing with a Visiting Professor fellowship in the Pasteur Institute in France. She then went on to complete a postdoctoral fellowship in the National Cancer Institute of the NIH, focusing now in keratinocyte in vitro culture. It was this expertise in skin cell culture that would eventually become the basis for her influential work with Dr. Anthony Atala, engineering ex vivo urethras and vaginas for children with congenital malformations. In 2010, this work was recognized with the Glaxo-Smith-Klein Surgery Award and the Mexican National Health awarded by Coparmex. Prof. López-Bayghen now applies her expertise in cell culture and molecular analysis to developing diagnostic methods for infertility, as well as new techniques to improve IVF procedures.

#### 3.3.3. Advances of Stereotactic Radiosurgery and Fractionated Stereotactic Radiotherapy: From the Brain to the Spine

Frighetto, L.

**Abstract**

Stereotactic radiosurgery (SRS) was developed based on the precise application of single high doses of radiation to an intracranial target. Initially designed for the treatment of functional diseases, SRS has achieved an important role in the management of a wide range of neurosurgical pathologies. Improvements in mechanical accuracy and computerized technology of modern linear accelerators as well as in software technology increased its the safety and precision. The excellent results achieved in cranial treatments have made spinal pathologies the next target for SRS. Its application to the spine was initially limited by technical aspects including the capability of a reproducible stereotactic positioning system, as well as a precise system for single dose near to the spinal cord. Further concepts of volumetric stereotaxis and image-guided surgery, also known as frameless stereotactic localization as well as intensity-modulated radiation therapy (IMRT) planning and delivery have brought spinal SRS to another level of confidence and precision, starting the modern era of stereotactic radiation dedicated to the spine and the whole body. The application of the important concepts of precision, fast delivery, high single dose, fractionates schemes, strict conformality, and frameless targeting used in cranial, spinal and whole-body pathologies will be discussed in this presentation.

**Biography**

Leonardo Frighetto is the scientific coordinator of the Stereotactic Radiosurgery Unit at Moinhos de Vento Hospital in Porto Alegre, Brazil. After finishing a fellowship at the Department of Stereotactic and Functional Neurosurgery at the University of California Los Angeles (UCLA), he participated in the development of one of the first Stereotactic Radiosurgery Units in Brazil. Moinhos de Vento Hospital is one of the few hospitals accredited by the Joint CommissionInternational (JCI) in the Country and is affiliated to Johns Hopkins Medicine International. He also served as the President of the Brazilian Society of Radiosurgery from 2012 to 2014. Nowadays, the department is a reference in south Brazil for Stereotactic Radiosurgery and Radiation Oncology.

### 3.4. Digital Medicine and Big Data

#### 3.4.1. Major Depressive Disorder, Prediction of Clinical Outcome after 4 and 8 Weeks of Citalopram/Escitalopram Therapy: A Data-Driven Machine Learning Approach

Weinshilboum, R.M.

**Abstract**

Pharmacotherapy response/remission rates in major depressive disorder (MDD) are subject to large inter-individual variability. In this study, we have used a data-driven machine learning approach to join validated measures of depression severity and metabolomic data at baseline to predict clinical response in depressed patients treated with citalopram/escitalopram with re-evaluation at 4 and 8 weeks. Specifically, we used the Quick Inventory of Depression Symptoms (QIDS) and Hamilton Depression Rating Scale (HDRS) to define clinical outcomes such as “response”, and “remission” at baseline, 4 and 8 weeks as well as concentrations of 31 plasma metabolites at baseline. These analyses resulted in the following major observations: (1) multivariate statistics established clear gender-dependent differences in SSRI response/remission; (2) unsupervised learning methods inferred, validated and quantified three classes or clusters of symptoms across the duration of the trial in both men and women; (3) the inclusion of biological measures (metabolomics) greatly enhanced our ability to predict clinical outcomes; and, finally, (4) we obtained patient-specific prediction accuracies of greater than 70% using supervised learning methods. Baseline plasma serotonin was the metabolite with the highest relative contribution to the accuracy of the predictive model—compatible with previous reports. In summary, we have demonstrated that the inclusion of baseline metabolomics data, one class of potential biomarkers—when combined with validated measures of MDD symptom severity prior to SSRI therapy—might help make it possible to better predict clinical outcomes in MDD patients treated with SSRIs and, perhaps, to provide novel insight into MDD pathophysiology.

**Biography**

Dr. Weinshilboum received B.A. and M.D. degrees from the University of Kansas, followed by residency training in Internal Medicine at the Massachusetts General Hospital, a Harvard teaching hospital, in Boston. He was also a Pharmacology Research Associate at the National Institutes of Health in Bethesda, Maryland, in the laboratory of Nobel Laureate Dr. Julius Axelrod. Dr. Weinshilboum began his affiliation with the Mayo Medical School and Mayo Clinic in Rochester, Minnesota, in 1972 where he is presently Professor of Molecular Pharmacology & Experimental Therapeutics and Internal Medicine as well as Mary Lou and John H. Dasburg Professor in Cancer Genomics Research. He also directs the Pharmacogenomics Program of the Mayo Center for Individualized Medicine and he is Co-Principal Investigator of the USA National Institutes of Health (NIH) Pharmacogenomics Research Network Center at the Mayo Clinic. Dr. Weinshilboum’s research has focused on pharmacogenetics and pharmacogenomics, and he has authored over 350 scientific manuscripts which address these topics. A major area of investigation initially was the pharmacogenetics of drug metabolism, with a focus on methylation and sulfation but, in recent years, his research has increasingly applied genome-wide pharmacogenomic techniques—especially to study the therapy of breast cancer-rather than candidate gene or candidate pathway-based approaches. Dr. Weinshilboum has been the recipient of many awards and honors including an Established Investigatorship of the American Heart Association, a Burroughs Wellcome Scholar Award in Clinical Pharmacology Award, the Oscar B. Hunter Award of the American Society for Clinical Pharmacology and Therapeutics, the Harry Gold Award of the American Society for Pharmacology and Experimental Therapeutics, the Catecholamine Club Julius Axelrod medal, the U.S. Food and Drug Administration William B. Abrams Lectureship Award, and the Edvard Poulsson Award from the Norwegian Pharmacology Society. He has also served on the Advisory Councils for two USA NIH Institutes, the National Institute of General Medical Sciences (NIGMS) and the National Human Genome Research Institute (NHGRI).

#### 3.4.2. Model-Based Unsupervised Learning Informs Metformin-Induced Cell-Migration Inhibition through an AMPK-Independent Mechanism in Breast Cancer

Wang, L.

**Abstract**

We demonstrate that model-based unsupervised learning can uniquely discriminate single-cell subpopulations by their gene expression distributions, which in turn allow us to identify specific genes for focused functional studies. This method was applied to MDA-MB-231 breast cancer cells treated with the antidiabetic drug metformin, which is being repurposed for treatment of triple-negative breast cancer. Unsupervised learning identified a cluster of metformin-treated cells characterized by a significant suppression of 230 genes (*p*-value < 2 × 10^−16^). This analysis corroborates known studies of metformin action: (a) pathway analysis indicated known mechanisms related to metformin action, including the citric acid (TCA) cycle, oxidative phosphorylation, and mitochondrial dysfunction (*p*-value < 1 × 10^−9^); (b) 70% of these 230 genes were functionally implicated in metformin response; (c) among remaining lesser functionally-studied genes for metformin-response was CDC42, down-regulated in breast cancer treated with metformin. However, CDC42’s mechanisms in metformin response remained unclear. Our functional studies showed that *CDC42* was involved in metformin-induced inhibition of cell proliferation and cell migration mediated through an AMPK-independent mechanism. Our results points to 230 genes that might serve as metformin response signatures, which needs to be tested in patients treated with metformin and, further investigation of *CDC42* and AMPK-independence’s role in metformin’s anticancer mechanisms.

**Biography**

Dr. Wang received an MD from FuDan University Medical School, Shanghai, China, followed by a Ph.D. in Pharmacology from Mayo Graduate School. She was trained in a leading pharmacogenomics laboratory and as a Clinical Pharmacology trainee supported by the Mayo NIH T32 Clinical Pharmacology Training Grant. After assuming a faculty position in Pharmacology at Mayo Clinic Rochester, her research focused on pharmacogenomics and precision medicine of cancer therapy using high throughput “omics” technologies to identify and understand biomarkers associated with variation in drug response. She is leading various functional studies of breast cancer pharmacogenomics using different model systems built in her lab including 300 genomically characterized human lymphoblastoid cell lines (LCLs) and patient derived xenograft models for breast cancer and castration resistant prostate cancer. She has been very successful in obtaining funding from the NIH, NSF, DOD and foundations such as the Breast Cancer Research Foundation, and she has published extensively in high impact journals. Beyond her research experience, she has been Co-PI for the Mayo multi-disciplinary NIH-Mayo Pharmacogenomics Research Network (PGRN) grant and the Mayo Clinical Pharmacology T32, and I am Co-Director of the Pharmacogenomics Program within the Mayo Center for Individualized Medicine (CIM).

#### 3.4.3. Virtual Surgery for Aortic Aneurysms

Bou-Saïd, B.

**Abstract**

The Abdominal and Thoracic Aorta Aneurysm (AAA and TAA) are cardiovascular disease that affects 6–7% of the Western population and their incidence increases with age. At least 90% of AAA and TAA come from atherosclerosis because of high cholesterol, inflammation, infection or tobacco... Most of these aneurysms are located near the bifurcation. The rupture of the aneurysm is a dangerous and fatal accident favored by arterial hypertension.

For over 50 years, open surgery was the only treatment of AAA. This is a major procedure with many risks of cardiac complications (myocardial infarction...), respiratory, bleeding, renal, infectious and colic (risk ofischemic colitis). However since 1991, a new mini-invasive surgical procedure has been introduced. This is an endovascular procedure that is to drag a stent through a release device of the femoral artery to the level of the aneurysm.

For this purpose, we have developed a numerical simulation tool to assist surgery. It contributes to the improvement of therapeutic endovascular procedures in terms of accuracy and optimizes the intervention strategy.

This tool takes into account: (1) the actual geometry bio-faithful reconstructed from preoperative clinical images on a specific group of patients with high tortuosity and calcification; (2) a local characterization of mechanical properties of the endovascular system; (3) a mapping of mechanical properties of soft tissues based on their degree of calcification (safe, calcified, thrombus); (4) hemodynamic with specific blood rheology; (5) FSI; (6) a projection of the real environment of the artery on the simulated model for each patient; (7) a pre-constraints; (8) a material and geometric non-linearity, a composite model for the wall artery...

**Biography**

Benyebka Bou-Said is Professor at INSA de Lyon and researcher at LaMCoS in a variety of subjects concerned with both fundamental and applied tribology, including hydrodynamics, fluid-structure interaction, rheology, tribochemistry and biomechanics (joint and vascular diseases). Pr. Bou-Said’s background in tribology encompasses bearings, dampers, magnetic devices, including both fluid film and fluid-structure coupling and biotribology. Pr. Bou-Saïd is the head of a research group involved in Tribology and Multiscale Approach to the Study of the Mechanics of Living Beings and Mechanisms: numerical and experimental models assuming the coupling of mechanics/physics/chemistry/biology. The main objective is: understanding the link between causes and consequences to optimization: diagnosis—prevention—Treatment of Pathology, Security and bPublic health. Pr. Bou-Saïd is Fellow ASME and STLE, guest editor for the Journal of Engineering Tribology (JET) and Tribology International and associate editor for Tribology Transactions and JET. He is chairman of the annual Leeds-Lyon International Tribology Conference. Pr. Bou-Saïd is listed Who’s Who in the World and has authored more than 140 papers. He has received the Tribology Gold Medal at the Japanese Tribology Conference Nagasaki October 2000 for his prospective work in the field of biotribology.

**Biography**

Paolo Milia, MD, Ph.D. is Chair of the Department of Neurorehabilitation and Robotic Area at Istituto Prosperius Tiberino, a position he has held since 2004. Dr. Milia is Vice President of International Society of Digital Medicine and Co. Editor’s in chief of the Journal Digital Medicine (DM). He completed his medical/surgical degree at G. D’Annunzio University in Chieti, Italy in 1998 and his residency in neurology in 2003. Subsequently, he was a neurologist, clinical research fellow, and lecturer at the University of Glasgow, Scotland. In 2007, he attained his Ph.D. in neurological research from the University of Perugia. Dr. Milia has taught physiopathology, nutrition, neurology, medical radiology, sports medicine, and sports neurology at the University of Perugia. In 2002, he received a European Stroke Conference Award for Excellence for his paper entitled “Serum Potassium and Stroke Outcome”. He has also received awards for his research on the effects of thiazides and related diuretics after stroke and for his work on the validity of the Italian version of the modified Rankin Scale (mRS), a measure of the degree of disability and dependence in individuals who have experienced a stroke. Dr. Milia is one of Europe’s leading researchers in the use of robotics to rehabilitate patients with a wide variety of neurological conditions, including spinal cord injury, stroke, multiple sclerosis, and Parkinsonism. Dr. Milia has authored numerous publications in his areas of expertise and is on the editorial staff of the journal of the Italian Society for the Study of Stroke (S.I.S.S.). Dr. Milia is the Chair of the Robotic Area with special interest in the use of exoskeletons in patients affected with spinal cord injury and stroke. At the moment he is involved in some research about the use of Ekso and Indego the two most important exoskeletons in the market. Originally from Jesi in the Marche, Dr. Milia now resides in Perugia, Italy.

#### 3.4.4. Title: Using Robots in Rehabilitation to Improve Outcome of Neurological Patients

Milia, P.

**Abstract**

Neurorobotics refers to the branch of science combining neuroscience, robotics, and artificial intelligence. It hence refers to all robots developed for interacting with or for emulating the nervous system of humans or other animals. A neurorobot can be developed for clinical purposes, for example neurorehabilitation or neurosurgery, or for studying the nervous system by emulating its properties, as it occurs for example in the walking robots based on central pattern generators. Many neurorehabilitation approaches and techniques have been developed to restore neuromotor function, aiming at the recovery of physiological movement patterns in patients with neurological pathologies. Neurorobots have the potential for accurate assessment of motor function in order to assess the patient status, to measure therapy progress, or to give the patient and therapist real-time feedback on movement performance. Ekso is a mobile exoskeleton that is intended for rehabilitation and mobility of individuals with neurological motor diseases. The device is designed to adjust easily to fit users ranging in height between 157 and 195 cm. The individualized fit is made using measurements at the thigh and shank to adjust length, and at the hips to adjust frontal plane width. The device is attached to the user’s torso with backpack style shoulder harnessing and a torso brace. A new Exoskeleton called Indego has been working in our Hospital too. Indego mirroring natural human movement, lean forward to initiate standing or walking and lean backward to stop and sit. An Indego app on the mobile device allows to control operation, change settings, and capture data without the need for tethered controls. Indego allows over ground training or personal mobility on a variety of surfaces both indoors and outdoors and we’re studying the utility of an exoskeleton used as home device.

#### 3.4.5. Enhancing Macrophage Drug Delivery Efficiency via Co-Localization of Cells and Drug-Loaded Microcarriers in 3D Resonant Ultrasound Field

Lee, Y.-H.

**Abstract**

In this study, a novel synthetic 3D molecular transfer system which involved the use of model drug calcein-AM-encapsulated poly (lactic-co-glycolic acid) microspheres (CAPMs) and ultrasound standing wave field (USWF) with frequency of 1 MHz and output intensity of 0.5 W/cm^2^ for macrophage drug delivery was explored. We hypothesized that the efficiency of CAPMs-mediated drug delivery aided by USWF can be promoted by increasing the contact opportunities between cells and the micrometer-sized drug carriers due to effects of acoustic radiation forces generated by USWF. Through the fluoromicroscopic and flow cytometric analyses, our results showed that both DH82 macrophages and CAPMs can be quickly brought to acoustic pressure nodes within 20 s under USWF exposure, and were consequently aggregated throughout the time course. The efficacy of cellular uptake of CAPMs was enhanced with increased USWF exposure time where a 3-fold augmentation (*p* < 0.05) was obtained after 15 min of USWF exposure. We further demonstrated that the enhanced CAPM delivery efficiency was mainly contributed by the co-localization of cells and CAPMs resulting from the application of the USWF, rather than from sonoporation. In summary, the developed molecular delivery approach provides a feasible means for macrophage drug delivery.

**Biography**

Dr. Yu-Hsiang Lee received his B.S. degree in chemical engineering department from Tunghai University (Taiwan, ROC) at 1998, the M.S. degree in chemical engineering department from University of Southern California (USA) at 2002, and the Ph.D. degree in chemical engineering department from University of Southern California (USA) at 2006. He held a research scientist position from 2006 to 2008 in Sierra Sciences LLC; a biotech company (Reno, NV, USA) right after receiving his Ph.D. degree in 2006, where he worked on drug discovery of telomerase-activating compounds for curing aging-associated diseases. Afterward, he joined Dental Research Institute at University of California Los Angeles (UCLA) as a postdoctoral fellow from 2008 to 2010, where he worked on salivary transcriptomic biomarkers discovery for early diagnosis of cancer and type II diabetes. He took the faculty position as an Assistant Professor in Graduate Institute of Biomedical Engineering at National Central University (Taiwan ROC) since 2010 and was promoted to Associate Professor in 2014. Now he is an Associate Professor in both Department of Biomedical Sciences & Engineering and Department of Chemical and Materials Engineering at National Central University (Taiwan ROC). Currently his research is focused on (1) development of theranostic agents for target photochemotherapy and diagnosis of breast cancer; (2) design of CO_2_-controlled photobioreactor system for fuel purification and enhancement of microalgae products; and (3) laminar shear stress-mediated endothelial & cancer cell biology.

#### 3.4.6. NCI-60 Data Exploration and Visualization

Wang, S.

**Abstract**

The NCI-60 human tumor cell line panel is an invaluable resource for cancer researchers, providing drug sensitivity, molecular, and phenotypic data for a range of cancer types. CellMiner is a web resource that provides tools for the acquisition and analysis of quality-controlled NCI-60 data. CellMiner supports queries of up to 150 drugs or genes, but the output is an Excel file for each drug or gene. This output format makes it difficult for researchers to explore the data from large queries. CellMiner Companion is a web application that facilitates the exploration and visualization of output from CellMiner. The application currently supports gene expression, microRNA expression, and drug activity data. Researchers can upload multiple files and then obtain a data matrix for original data, a matrix of z-scores for this data, a heatmap of using the z-score matrix, and a dendrogram of the hierarchical clustering. Researchers can interactively change multiple parameters for data analysis and visualization, making CellMiner companion a useful tool for data exploration and hypothesis generation, further increasing the accessibility of NCI-60 data.

**Biography**

Dr. Sufang Wang is a recognized expert in Bioinformatics. She obtained her Ph.D. from Purdue University. During her Ph.D., she started work on next-generation sequencing, including evaluation of de novo assembly programs and analyze RNA-seq data for plant and human cancer. In 2017, when she graduated, she come back to China and started her faculty career in Northwestern Polytechnical University. Currently, her major research interest is regulation and mechanisms of immune cells under microgravity condition.

### 3.5. Tissue Repair and Regeneration Medicine

#### 3.5.1. Transformation of Mesenchymal Stem Cells (Mscs) into Mesangial Cells (Mcs) Represents an Important Process in the Stem Cell Repair and Regeneration

Teng, J.

**Abstract**

Many investigators have supported the idea that mesenchymal stem cells (MSCs) participate in the process of repair/regeneration exclusively by providing paracrine factors that enabled the process. Using a model of mesangial damage induced by glomerulopathic immunoglobulin light chains (G-LCs) and then repaired or regenerated by MSCs in AL-amyloidosis, the role of MSCs in the process was investigated.

In-vitro and ex vivo experimental platforms were used to address the issue. The in vitro 6 dimensional (6D) live cell imaging system was used to observe the damage of mesangial cells (MCs) and the alteration of the mesangial matrix incubated with G-LCs. In the ex vivo model, G-LCs were perfused through the renal artery. The respective lesions were reproduced in both platforms. Then, tagged MSCs were introduced. Immunofluorescence, immunohistochemistry and electron microscopy were used to evaluate samples obtained at different time frames. Stains for smoothelin, muscle specific actin, smooth muscle actin, CD29, and 68 were used to monitor phenotypic transformation of MSCs in the process of regeneration.

Our results showed that MSCs initially transformed from an undifferentiated to a macrophage phenotype to clear the damaged mesangial areas where transformed MSCs phagocytosing cellular debris resulting from apoptotic mesangial cells and damaged matrix elements of amyloid fibrils. Following the cleaning process, MSCs acquired morphologic and immunophenotypic characteristics of MCs as they proceeded to lay down new mesangial matrix.

MSCs manifest great plasticity as they proceed to repair the damaged mesangium in both our models. The fact that they transform to a macrophage phenotype followed by transformation to MCs allows them to perform different crucial functions during the process of regeneration. The restored mesangium is possible as new MCs derived from MSCs are able to reproduce the normal mesangium.

**Biography**

Dr. Jiamin Teng is a professor in the Department of Pathology and Translational Pathobiology, Louisiana State University Health Sciences Center in Shreveport (LSUHSC), USA Dr. Teng’s research has been focusing on glomerulosclerosis of kidney damage and the stem cell regeneration, which can be translated to diabetic nephropathy due to similarities in pathogentic mechanisms involved. Dr. Teng’s laboratory has developed unique experimental models such as in vitro live cell micromanipulation model, in vitro 6 dimensional live cell observation models, ex vivo kidney perfusion model, and in vivo model for the kidney glomeruli damage and reparation. These models have well served for the purpose of kidney pathogenic and regeneration.

#### 3.5.2. Generic Glomerular Damage Viewed through a Unique Experimental Model: Understanding the Repair that Needs to Be Accomplished. What We Know and What We Need to Find Out

Herrera, G.

Mesangial injury represents a crucial event in the pathogenesis of light chain-associated glomerulopathies in patients with plasma cell dyscrasias. The glomerulopathic light chains interact with mesangial cells where purported receptors regulate the downstream cellular mechanisms that will be activated and result in glomerular alterations. The physicochemical and conformational characteristics of the abnormal light chains are primarily responsible for the downstream events affecting the mesangial milieu.

**Abstract**

Different light chains are responsible for two diseases with diametrically opposite mesangial alterations: Light chain deposition disease which results in the expansion of the mesangium due to accumulation of matrix proteins not present in the normal mesangium and AL (light chain-associated) amyloidosis where the native mesangial matrix is replaced by fibrils (amyloid). In both cases there is enhancement of mesangial cell apoptosis and the altered mesangium has a marked decreased of mesangial cells, most undergoing apoptosis.

The repair of the damaged mesangium is difficult due to the absence of enough mesangial cells that can participate in the process and also the damage can be so extensive that the intrinsic processes that are available for repair (i.e., recruitment of stem cells from bone marrow and precursor stem cells in renal niches) cannot effectively carry out the recovery. Introducing exogenous mesenchymal stem cells represents a novel therapeutic avenue that has been experimentally tested with promising results. Glomerular repair is hindered by how much of the glomerulus has become sclerosed and how to re-establish capillary walls and spaces in segmentally sclerosed/hyalinized glomerular areas. Disposing of the sclerotic material represents a major challenge for repair mechanisms to be able to restore homeostasis.

In-vitro, ex vivo and in vivo animal models have been created to study these disorders providing excellent platforms to elucidate pathogenesis and provide insightful information that can be translated to the practice of renal pathology, as the in vitro and in vivo platforms corroborate each other. The presentation will address how mesangial injury occurs in LCDD and AL-amyloidosis as examples of generic mesangiopathies.

**Biography**

Dr. Herrera is currently Chair of Pathology and Laboratory Director of LSU Health Shreveport, a position he has held from 1996 to 2006 and since 2012 until now. He is an internationally renowned nephropathologist with over 40 years of experience in renal pathology. Having received his Doctor of Medicine at the University of Puerto Rico, he has over 30 years of research on renal damage associated with multiple myeloma. He has served as Professor and Chair at St. Louis University as well as Director of Surgical Pathology at both the University of Alabama and the University of Mississippi. Dr. Herrera sits on multiple national panels and has been the invited guest lecturer in over 300 domestic and international venues. He has authored more than 300 articles and chapters. He is the recipient of a grant from the Amyloidosis Foundation to study glomerular repair in AL-amyloidosis using mesenchymal stem cells.

#### 3.5.3. Mesangial Repair by Mesenchymal Stem Cells: Where We Are

Turbat-Herrera, E.

**Abstract**

The study of stem cells is a growing field in which these are used to repopulate; therefore, repair damaged tissues. In the kidney, studies of stem cell repair have been conducted for repair of damaged tubules but very little has been published about glomerular repair.

Our laboratory has been engaged in the study of mesangial repair after injury induced by using mesenchymal stem cells (MSC) to repopulate the damaged mesangium and differentiate into mesangial cells. Our unique experimental model of mesangial injury has allowed us to explore the use of exogenous stem cells for mesangial repair of the glomerular damage produced by two different physicochemically and, therefore, conformationally (stoichiometrically abnormal) different light chains.

In vivo and in vitro platforms have been used to observe mesangial damage and repair. The sequence of events that leads to mesenchymal stem cells identifying the site of glomerular damage, eliminating the remnants of the damaged mesangium such as apoptotic cells and extraneous matrix (LCDD and AL-amyloid) and to differentiating into mature mesangial cells which then lay down new mesangial matrix has been elucidated. Part of our in vitro studies includes our observations utilizing a six dimensional (6D) live cell culture system by which the actual glomerular repair was observed and digitally recorded for up to 14 days. By this method the process of mesenchymal stem cells clearing mesangial debris and differentiation into mesangial cells can be analyzed in detail.

Each of the platforms will be presented to show their advantages and limitations. By studying the information obtained by each model system we have been able to better understand how exogenous mesenchymal stem cells can participate in the repair of the glomerular mesangium.

In the process of mesangial repair, mesenchymal stem cells exhibit marked plasticity which allows them to function first as macrophages to clean up the damaged mesangium, and then in the advanced phase of the repair process, to transform into and function as mature mesangial cells.

**Biography**

Dr. Turbat-Herrera is currently Professor of Pathology, a position she held from 1996 to 2006 as well as from 2012 to present and of Cell Biology and Anatomy, and the Feist-Weiller Cancer Center at LSU Health Shreveport, since 2012 until present. She is well known and internationally recognized for her work as a cytopathologist and her work on Fine Needle Aspiration of tumors and their diagnosis with the use of electron microscopy. In addition, she has expertise in Gynecologic and Genitourinary Pathology. She has given over 100 major lectures at national and international meetings and published nearly 100 peer reviewed articles and book chapters. Her work in cytopathology along with her residents and fellows has been awarded with three Awards from the Papanicolaou Society. Dr. Turbat-Herrera continues to practice and do research at Louisiana State University Health and is currently the director of the Tumor and Serum Repository and the Histology facilities of LSUHSC and the Feist-Weiller Cancer Center as well as Director of Cytopathology Training Program.

#### 3.5.4. Three-Dimensional Bioprinting for Cartilage Regeneration

Chen, H.H.

**Abstract**

Articular cartilage is hyaline cartilage and plays an important role in joint activities through bearing the mechanical load or lubricating joints. Unlike most tissues, articular cartilage does not have blood vessels, nerves, or immune response, and shows limited capacity for self-repair after degeneration or injury. Current approaches to treat articular cartilage lesions are often result in fibrous repair tissue, which may lead to degenerative changes and arthritis. 3D bioprinting is a promising biofabricationapproach for cartilage regeneration and overcome many limitations of current methods of articular cartilage repair. After we hadsuccessfully developed a series of products, including EHS Hydrogel, Special-coated cell culture plates and Feeder Protein Mix, and published two papers for stem cell expansion and differentiation into chondrocytes. We started to hit the last mile of stem cell application on cartilage regeneration. We started our collaboration with BioBots, a 3D Bio-printing company and introduce their 3D Bio-printers into Chinese market. Considering that the 3D bioprinting materials (Bioinks) are the key for 3D bioprinting, we start to develop and manufactureLAP (blue light initiator), and a variety of Bioinks, e.g., GelMA, HAMA, CSMA and ALMA. With our bioinks, we can print out biological scaffold with life chondrocytes in standard shapes, e.g., ring, star, column, and any irregular shape, e.g., nose-shape, ear-shape.Chondrocytes in the frame can grow well in vitro and in vivo, secrete col II, GAG and chondroitin sulphate, which replace those bioinks, and help the printed tissue regain the biomechanics strength of cartilage. We also are developing “Biopen”, which enables the deposition of living cells and biomaterials in a manual, direct-write fashion, and is welcomed by orthopedic surgeons, who like to repair and fix cartilage lesions on site.

**Biography**

Dr. Harry Huimin Chen, is the Founder and CSO of StemEasy Biotech Ltd., Jiangyin, China. He graduated from Sichuan Medical University in 1983, and owned his Ph.D. at Beijing Medical University (major of Sports Medicine/Molecular Biology) in 1990. Dr. Chen studied as a post doctorat Harvard Medical Schoolin 1991, became their Instructor of Medicine in 1994, and published more than 20 articles on peer-reviewed journals during 1990–1998. He entered the pharmaceutical industry in 1999, and was a senior scientist in Wyeth R&D center at Cambridge, MA during 1999–2005, and the head of General Management Department of GSK R&D China during 2008–2014. Dr. Chen was the winner of Wuxi “530 Plan” Talent-recruiting Program 2007, and found the StemEasy Biotechnology Company in same year. StemEasy mainly focuses the development of stem cell-related products and their applications, meanwhile based on the company’s expertise, provide technical services on stem cell and 3D bioprinting. In 2009, StemEasy was granted an Innovation Fund from Science and Technology Bureau in Jiangsu Provinceand matched fund from Jiangyin municipal government, the projectnumber is SBC200910486. In 2014, with the support from Dr. Wei Yuquan, academician of the Chinese Academy of Sciences, StemEasy set up the Academician Workstation and Stem Cell Engineering Research Center at Jiangyin. In March 2015, StemEasy established a cooperation relationship with a US 3D bio-printing company—BioBots and started to introducetheir 3D Bio-printing technology and products into Chinese market. In 2016, StemEasy teamed up with top 3 universities at Nanjing and Medical Engineering Research Institute of Chinese Academy of Sciences, was granted a funding from Jiangsu Key Technology RD Program (BE2016010), entitled “Development of key technologies and complete sets of equipment for 3D printing of Hip Prosthesis System toward precision medicine”.

#### 3.5.5. Stem Cell and Tissue Engineering

Purwati

**Abstract**

Stem cell have a characteristic are self-renewal and differentiation. Sources of stem cell are from autologous (if taken from their own bodies) and allogenic (if taken from outside). Kind of sources of stem cell are embryonic stem (ES) and adult stem cells that are lineage-restricted (multipotent) and generally referred to their tissue origin (mesenchymal stem cell, adipose-derived stem cell, endothelial stem cell, dental pulp stem cell, etc.). Cell manufacturing considerations are prevent transmission of infectious agent from donor or during cell product manufacturing, characterization of the product, stability of the product, how to measure product potency, current good practice, and safety concern of cell therapy which are cell differentiation, cell migration, potential uncontrolled cell proliferation or tumorogenesis, immunogenicity, graft vs. host effects, gene modified cell, and contamination. In some cases, stem cell cannot use alone but must be combine with scaffold, it called tissue engineering. Tissue engineering evolved from the field of biomaterials development and refers to the practice of combining scaffolds, cells, and biologically active molecules into functional tissues. The goal of tissue engineering is to assemble functional constructs that restore, maintain, or improve damaged tissues or whole organs. This process has been used to bioengineer heart, liver, lung, and kidney tissue. This approach holds great promise for using scaffolding from human tissue discarded during surgery and combining it with a patient’s own cells to make customized organs that would not be rejected by the immune system. Many different materials (natural and synthetic, biodegradable and permanent) have been investigated. New biomaterials have been engineered to have ideal properties and functional customization: synthetic manufacture, biocompatibility, non-immunogenicity, nano-scale fibers, low concentration, resorption rates, etc. Examples of these materials are collagen and some polyesters.

**Biography**

Dr. Purwati has finished in general practitioner from Airlangga University in 1997, has completed in internal medicine. Specialist in 2008 from Airlangga University also and taken Doctoral program in Airlangga University 2010–2012. Interest in stem cell field from 2008, be secretary of stem cell laboratory of Airlangga University and also secretary of Surabaya Regenerative Medicine Centre. 2015 be a chairman of Stem Cell Research and Development Centre Universitas Airlangga Surabaya Indonesia. Have almost 50 publication in journals, papers, and seminar.

### 3.6. Cell Engineering

#### 3.6.1. What Does Mechanical Force Affect Eph/Ephrin Signalling in Periodontal Ligament Cells?

Yang, Y.

**Abstract**

Eph receptor tyrosine kinases are a large family of transmembrane proteins with an extracellular globular domain that enable them to bind with N-terminal site of ephrin ligands. The combination of ephrin-Eph activates the single cytoplasmic kinase region of Ephs and subsequently transduces the bidirectional signals into both receptor- and ligand-expressing cells. The forward signal transduction through Ephs and the reverse signaling via ephrins trigger a number of downstream cascades that regulate the bioprocess among cells and tissues. The ephrinA2-EphA2 and ephrinB2-EphB4 system has been reported as important mediators in bone cell communication and skeletal development. The interaction between ephrinA2 and EphA2 generally promotes osteoclastic differentiation and suppress osteoblastic differentiation, while that between ephrinB2 and EphB4 functions on the opposite side thus benefit bone formation. Immediately after the force applied to the tooth, the periodontal ligament (PDL) tissue is compressed on one side and is stretched on the other side. Tooth movement towards the pressure area is the result of mechanical force-induced alveolar bone and PDL tissue remodeling. Therefore, PDL cells are the target cells of the orthodontic tooth movement. We will review our findings and present what mechanical force affects Eph/ephrin signaling in periodontal ligament cells, in an attempt to better understand the mechanism of bone remodeling under mechanical loading.

**Biography**

Dr. Yanqi Yang (BDS; MOrth RCS Ed; Ph.D.; FDS RCS Ed) is the Clinical Associate Professor in Orthodontics, Discipline Coordinator, Postgraduate and Undergraduate Programme Director in Orthodontics, and the past Deputy Convenor of Biomedical and Tissue Engineering Research Group in Faculty of Dentistry, the University of Hong Kong. Her research interest is bone biology and mechanism of orthodontic tooth movement. She is the member of World Federation of Orthodontists, Hong Kong Society of Orthodontists, Hong Kong Dental Association, International Association of Dental Research, American Association of Orthodontists and European Orthodontic Society. She is the reviewer of 20 international refereed journalssuch as International Journal of Dentistry, Journal of Investigative and Clinical Dentistry, The Angle Orthodontist, Scientific Research and Essays, European Journal of Orthodontics, Oral Health and Preventive Dentistry, Regulatory Peptides, etc.

#### 3.6.2. Recent Research on Surface Modification of Cardiovascular Biomaterials Inspired from the In Vivo Endothelial Microenvironment

Li, J.

**Abstract**

Cardiovascular disease is generally accepted as the leading cause of morbidity and mortality worldwide, and lots of people suffer from atherosclerosis and thrombosis every year. To treat these disorders and prolong the patients’ life, the cardiovascular devices have been developed and applied clinically. Nevertheless, thrombosis and hyperplasia at the site of implanting cardiovascular devices are recognized as long-term problems in the practice of interventional cardiology. Although the drug-eluting stents make excellent contribution to treat early thrombosis and inflammation, the loading drugs also delay vascular healing and re-endothelialization, leading to high risk of late thrombosis and hyperplasia. Recent researches show that vascular endothelial functional reconstruction at the lesion can significantly reduce the late thrombosis and hyperplasia, and this process is closely related to the endothelial cells growth microenvironment, including the pericytes environment, blood flow shear stress and the extracellular matrix microenvironment. Therefore, the in situ construction of endothelial cells growth microenvironment is of great importance for improving the biocompatibility of the cardiovascular stents. Here, we introduced several novel strategies on surface modification of cardiovascular biomaterials in our research group and discuss the prospects for extending use of the in situ construction of endothelial cells growth microenvironment in designing the next generation of vascular stents.

**Biography**

Dr. Jingan Li is an associate professor at Zhengzhou University, China. He received his M.Sc. degree in Biochemistry and Molecular Biology (2009) and Ph.D. in Materials Science and Engineering (2013) from Southwest Jiaotong University, China. Then, he continued his work as Postdoctoral Fellow in Institute of Biomaterials and Surface Engineering, Southwest Jiaotong University (2014). He was awarded “China-Korea Young Scientist Award” at Chinese Biomaterials Congress 2015 and “Outstanding Reviewer Award” by the Journal—Materials Science and Engineering C (2015). He was invited as a session Chair of “Biomaterials as stem cell microenvironment” session at the 10th World Biomaterials Congress 2016. He was awarded by “International Association of Advanced Materials Scientist Medal (IAAM Scientist medal) for the year 2017” by International Association of Advanced Materials for notable and outstanding contribution in the field of “Advanced Materials Science and Technology” in Singapore. His current research is particularly focused on surface modification of cardiovascular biomaterials and degradable biomaterials.

#### 3.6.3. Understanding Stem Cell Fate through Live Cell Imaging and Single Cell Gene Expression Analysis

Nordon, R.E.

**Abstract**

Understanding of how extrinsic and intrinsic factors direct stem cell fate is central tothe development of in vitro systems to study disease and regenerative therapies. In vitro differentiation of stem cells into tissue-specific cell types will have great utility for transplantation therapies such as blood stem cell transplant and cellular immune therapies. Our lab focuses on developing imaging andlab-on-a-chip to track stem cell fates, and to relate their differentiation path to single cell gene expression analysis. We are currently using a mix of technologies to study (a) development of blood from haemogenic endothelium (b) cytotoxic mechanisms for chimeric antigen receptor T-cell (CART) killing of cancer cells and (c) cellular response to cardiac repair following myocardial infarction. We have evaluated rapidly advancingmicrofluidic commercial technologies for high-throughput, single cell gene expression analysis (Fluidigm™ and drop RNA seq). Single cell gene analysis allows one to characterise complex heterogeneous cellular systems in extraordinary detail: Stages of cell development can be further refined using single gene expression signatures. Furthermore, cellular communicationscan be characterised by bioinformatic analysis of cell-specific, secretome and receptor-signalling pathway genes.

**Biography**

Dr. Robert Nordon graduated from the University of New South Wales in 1986 with an MBBS and received his Ph.D. in the field of Biomedical Engineering in 1994. He undertook postdoctoral research in The Terry Fox Laboratory in Vancouver, Canada, in experimental haematology, before returning to UNSW in 1997 to continue postdoctoral studies in the Graduate School of Biomedical Engineering on an ARC Australian Post-Doctoral Fellowship, and he has been Senior Lecturer in the same department since 2006. Dr. Nordon is considered a National authority in the area of mammalian cell bioreactors for clinical therapies. He is the inventor of a hollow fibre bioreactor that was commercialised by a USA blood component therapy company, Ceridian BCT (formally Gambro, BCT). Dr. Nordon has also made significant contributions in the field of analytical cytology, which is highly relevant to this proposal. He has developed experimental and theoretical methods for analysis of multipotent cell differentiation, which is now widely cited in the literature. He is currently refining methods for single cell fate mapping using “lab-on-a-chip” devices. His role in the Stem Cells Australia will be to collaborate with the Harvey laboratory in single cell real time analysis of cardiac stem cell growth and differentiation using microfluidics technology.

#### 3.6.4. Reinforcement of STAT3 Activity Reprogrammes Human Embryonic Stem Cells to Naïve-Like Pluripotency

Chen, H.

**Abstract**

LIF/STAT3 signaling is a hallmark of naïve pluripotency in rodents, whereas FGF2 and activin/nodal signaling is required to sustain self-renewal of human pluripotent stem cells in a condition referred to as the primed state. It is unknown why LIF/STAT3 signaling alone fails to sustain pluripotency in humans. Here we show that the forced expression of the hormone dependent STAT3-ERT2 in combination with LIF and tamoxifen allows human embryonic stem cells to escape from the primed state and enter a new state designated as TL, which is characterized by the activation of STAT3 target genes and long-term self-renewal in FGF2-and feeder-free conditions. Moreover, when TL cells are propagated in 2i/LIF conditions, they acquire growth properties, a gene expression profile, and an epigenetic landscape closer to those described in mice. Together, these results show that temporarily increasing STAT3 activity is sufficient in reprogramming human embryonic stem cells to naïve pluripotency.

**Biography**

Dr. Hongwei Chen is a PI and Deputy Director of Institute of Rheumatology and Immunology, The Affiliated Drum Tower Hospital of Nanjing University Medical School, China. He obtained his Ph.D. from Kunming Institute of Zoology, the Chinese Academy of Sciences in 2008. Dr. Chen did his first round postdoc training at Stem Cell and Brain Research Institute, INSERM in France during 2008–2014. Then he worked for Wellcome Trust Sanger Institute in Cambridge, UK, as postdoc and staff scientist afterwards till July 2017. As a stem cell scientist, his research interest currently covers distinct pluripotent status in mammalian cells, neural differentiation, and stem cell therapy for autoimmune diseases.

#### 3.6.5. MRISim: A Software for Virtual MRI Simulator

Wang, H.

**Abstract**

Magnetic resonance imaging (MRI) is a complex process, incorporating quantum physics, electronic information science, mathematics, statistics, imaging, etc. Significant efforts have been invested to address MRI simulation to make the learners gain a better understanding of the technology.

For any sample, the virtual data acquisition in one phase encoding and filling the K space by NE encodings, then reconstruct image from K space by FFT . Based on it we developed a software named MRISim t simulation results similar to those obtained with a real instrument, but in a more time- and cost-efficient manner. The software including MRISim interface modules: (a) pre-scan, (b) auto-tuning and matching and (c) imaging and artifacts analysis (Figure 1). Using MRISim, operators can perform more than 20 experiments, MRISim can also incorporate the simulation of more than 11 artifacts in MRI experiments, such as truncation, aliasing, chemical shift, RF interference, sparking etc., through simulating the corresponding errors and/or applying various hardware or software parameters. MRISim provides an accurate and efficient tool for simulating MRI process suitable for training MRI operators, engineers, and graduate students. The development of a software package that provides virtual magnetic resonance imaging (MRI) data acquisition and image reconstruction (MRISim) was introduced. MRISim is a time- and cost-efficient tool for simulating MRI process and results on a computer. It has proven to be efficient and reliable tool for the training of MRI operators, engineers, and graduate students.

**Biography**

Professor Hongzhi Wang is a researcher on NMR relax-meter and MRI of the Key NMR laboratory of East China Normal University Now. He have been as vice director of medical imaging college of shanghai medicine and health university for 3 years. And from 2014 to 2015, Dr. Wang has been studied as a visiting scholar in Harvard medical school and Massachusetts General hospital. Dr. Wang has published over 50 papers on the subject of MRI and NMR and MRR for hardware and software design and application. He has held more than 20 research project on instrument design and three kind of products has been sold to customer. He is a recognized expert in the MRI experiment teaching, and published 4 textbooks about MRI experiment. His education list is: Beginning date September 1994, and end date July 1998, Graduated from Zhongnan Institute of Technology (now University of South China) in 1998 with a Bachelor of Engineering. Beginning date September 2002, and end date July 2005, Graduated in Measurement Technique and Measurement Instruments from Shanghai University in 2005 with a Master of Engineering. Beginning date September 2007, and end date May 2011. Graduated in Radio Physics from Shanghai Nuclear Magnetic Resonance Kay Laboratory, East China Normal University in 2011 with a Doctor of Science.

#### 3.6.6. Three-Dimensional Simulation of a Red Blood Cell in Shear Flow

Huang, W.-X.

**Abstract**

I will present an improved penalty immersed boundary (pIB) method for 3D simulation of the fluid-membrane interaction. The membrane deformation takes full account of the bending and twisting effects as well as the stretching and shearing effects. Hence, the method of subdivision surfaces is adopted to generate the mesh of membrane and the corresponding shape functions, which are required to be *C*_1_ continuous. Using the proposed pIB method, the deformation of a biconcave circular disk as a model of the red blood cell in a linear shear flow is studied in detail. In our simulation, the swinging motion is observed due to the shape memory effect. By decreasing the dimensionless shear rate or increasing the reduced bending modulus, the swinging motion is transited into the tumbling motion.

**Biography**

Dr. Wei-Xi Huang received his bachelor degree and master degree from Tsinghua University in 2001 and 2004, respectively, and he completed his Ph.D. at KAIST in 2009. In 2010, Huang entered the school of aerospace engineering of Tsinghua University as a lecturer, where he is now serving as an associate professor (2012–now). Huang’s current research focus is on numerical study of turbulence control and development of novel schemes for turbulent drag reduction. Huang is also interested in computational biofluid mechanics. He has been developing computational methods for fluid-flexible body interactions, with the goal of simulating and obtaining physical insight into problems from biomechanics. Huang received China National Science Funds for Excellent Young Scientists in 2013. Huang is currently an editorial board member of Journal of Mechanical Engineering Science, and he is also a member of the international exchange and cooperation committee of Chinese Society of Theoretical and Applied Mechanics.

#### 3.6.7. Low Back Disorder: Research Method and Associated Sports Activity

Shan, X.

**Abstract**

Low back disorder (LBD) is one of serious public health problems in many industrialized countries. It has caused severe disruption for sufferer’s quality of life through limiting his/her professional, quotidian and laboural tasks. LBD has been highly concerned in industry due to replacing and qualifying staff as well as in National Health Services due to medicating LBD and premature retirement. There are many aspects in detecting LBD. Experimentally, electromyography (EMG) is one of the most important methods in evaluation LBD. EMG signals are detected on back muscles such as erector spinae to test the flexion relaxation phenomenon (FRP), or the co-activation between different muscles during anterior trunk flexion-extension exercise, which is thought to be associated with LBD. Epidemiologically, trunk bending as well as lifting activities is thought to be a risk factor for the development of LBP. Therefore, there are several sports activities, such as static compressive loading, trunk axial twisting, and trunk forward bending, which may be associated with the development of LBD. In addition, large load lifting during forward trunk bending could elicit spasms on back muscles which implicate some damage on ligaments in low back area.

**Biography**

Professor Xinhai Shan is the professor at Biomechanics Laboratory, College of Physical Education, Shandong normal university, Jinan, China. He received his BSc in Fluid Machinery from Jiangsu University, MSc in Engineering Mechanics from Tsinghua University and Ph.D. in Sport Biomechanics from Beijing Sport University in China. He worked as a research assistant professor at Sports Biomechanics Laboratory in Jiangsu Research Institute of Sports Science from November 1989 to July 1998. He worked as a visiting professor in Bioengineering Division, School of Medicine, University of Colorado Denver from September 2009 to March 2010. He has published more than 30 papers on many areas in Sports Science. His main research interests now are sport injuries and low back disorder.

#### 3.6.8. Cloning of Hair Follicle and the Difficulties within

Wu, Z.

**Abstract**

Hair transplantation with healthy follicles was proven possible by Dr. Okuda in the 40s, followed by the first successful attempt in culturing Hair follicular cells in vitro in the 1960s by Colin Jahoda. Since then many attempts has been made toward cloning functioning hairfollicles to save people from hairloss.

After the year 2000 several independent clinical trials conducted by several groups showpromising yet confusing results. Canterbury Pharmaceuticals has been working on haircloning on animal models and has interesting discoveries to share.

**Biography**

Zilong Wu is the research director of Canterbury Pharmaceuticals working on hairfollicular cloning and on developing protein vectors targeting neuronal cells. After graduatingfrom University of Auckland with MSc degree he has being working on biomedical researchand development in the industry. In addition to follicular regeneration and protein drugresearch, he also works on establishing chemically defined serum free stem cell culture mediafor medical use.

#### 3.6.9. Neuroprotective Effect of Artemisinin and Its Underlying Mechanism

Zheng, W.

**Abstract**

Artemisinin, also known as qinghaosu (Chinese: qinhaosu). It is an antimalarial drug derived from qinghao. In the present study, we found that artemisinin promoted the survival of various cell types such as PC12 cells and primary cortical cultured neurons from oxidative insults. Pretreatment of PC12 cells with artemisinin significantly suppressed SNP/H_2_O_2_-induced cell death by decreasing the production of intracellular reactive oxygen species (ROS), preventing the decline of mitochondrial membrane potential, restoring abnormal changes in nuclear morphology and reducing LDH and caspase 3/7 activities. Western blot analysis showed that artemisinin was able to stimulate the phosphorylation/activation of extracellular regulated protein kinases (ERK) kinase, AMPK and CREB while had no effect on the Akt pathway. In addition, ERK and AMPK signaling pathway inhibitors attenuated the protective effect of artemisinin whereas the PI3K inhibitor LY294002 had no effect. Taken together, these results suggested that artemisinin is a potential neuroprotectant which is able to suppress various oxidative stresse’s induced neuronal cell death via the activation of ERK/AMPK signaling. Our results offer support for the potential therapeutic application of artemisinin to prevent neuronal degenerative disorders. Supported by NFSC (31771128 and 31371088), FDCT (021/2015/A1 and 016/2016/A1), SARG and MYRG2016-00052 from University of Macau.

**Biography**

Dr. WenHua Zheng, Professor, Principle Investigator in Faculty of Health Science, University of Macau, leading a group of scientists working on aging and neuronal degenerative disorders including Alzheimer’s disease and degenerative retinal diseases; New functions and downstream targets of FoxO; Protective effect of Artemisinin and new drug developments. He is the Editor-in-Chief for the Journal of Clinical & Experimental Pharmacology, a Lead Guest Editor and Editor for several journals. Reviewer for NSFC, Poland and CIHR in Canada and an Adjunct Professor at RMIT University in Australia. Dr. Zheng has published >100 papers which have been cited over 3400 times.

#### 3.6.10. Hypoxia Inducible Factor (HIF2a) Regulates Muscle Stem Cell Physiology

Xie, L.

**Abstract**

Muscle stem cells (MuSCs) are essential to postnatal muscle repair and hence long-term maintenance of skeletal muscle function. Accumulating evidence indicates that the niche factor plays an essential role in maintaining SCs homeostasis, including quiescence, activation, proliferation and differentiation. A recent study shows that hypoxic culture promotes myoblast self-renewal and increases myoblast transplantation efficacy. Here, our data demonstrate that stem cell niche hypoxia stabilizes only HIF2α but not HIF1α, as a key transcription factor regulating MuSCs homeostasis. Meanwhile, HIF2α expression in MuSCs is dynamically changed during injury-induced muscle regeneration. Furthermore, SC-specific disruption of HIF2α expression results in spontaneous activation and proliferation. By contrast, overexpression of mutated HIF2α in SCs leads to promoting SCs quiescence/self-renewal, suppressing SC proliferation and differentiation. We also prove that transient overexpression of HIF2α in myofiber-associated SCs improve the engraftment of transplanted SCs into injured TA muscle. Notably, SC-specific deletion of HIF2α accelerates the muscle regeneration. We also demonstrate that pharmacological inhibition of HIF2α function during muscle regeneration promote SCs proliferation, and accelerate regeneration progress without affecting the total number of SCs in niche, fiber size and fiber type after regeneration. Therefore, in this study, our data first-time demonstrate that HIF2α is an essential transcription factor to regulate MuSCs homeostasis and enhance the engraftment of transplanted MuSCs into the injured muscle. On the other hand, HIF2α also could be a potential therapeutic target during muscle regeneration.

**Biography**

Dr. Liwei Xie obtained double Bachelor degrees from both Electrical Engendering in 2007 from Xi’an University of Science and Technology, and Biological Sciences in 2009 from State University of New York University at Buffalo. Later, he moved to University of Florida, joined Dr. James F. Collins lab at the Food Science and Human Nutrition Department in 2009 and obtained his Ph.D. degree in Human Nutrition in 2013. His Ph.D. research mainly focus on the regulatory mechanisms of Hypoxia Inducible Factors (HIFs) on intestinal mineral absorption and systemic homeostasis. After the Ph.D. training, he spent one year in the Dr. Yatrik Shah lab at the University of Michigan medical school, where he extended his research to physiological importance of HIFs on intestinal health and diseases. In 2014, he joint Dr. Hang Yin lab at Center for Molecular Medicine of University of Georgia. In the past three years, most studies focus on elucidating the physiological importance and mechanistic function of HIFs in regulating muscle stem cells physiology. Liwei has broad research training and interest in human physiology and diseases. His future research will focus on understanding the molecular mechanisms of intestinal mineral absorption and systemic homeostasis, and muscle as well as adipocyte stem cell physiology. Successfully completing these studies will provide us broad understanding and translational implication in treating human health related problems like iron deficiency-related diseases, muscular dystrophy and metabolic diseases, e.g., obesity and type 2 diabetes.

#### 3.6.11. Impact of Deploying a Genetic Approach to Stem Cells Opens-Up New Facets in the “Blank Slates” of Our Body

Bhojwani, J.

**Abstract**

Since the dawn of the Post-Genomic era (25 years back), applying a genetic approach to solving various intricate problems/issues in research has taken-off even more swiftly than ever before. Spatio-temporal cues defined for certain critical components in a particular developmental pathway (involved in causing/progression of certain disease) provide a firm basis for detecting the order, hierarchy and “switching-off or on” of genes that regulate it. The various time-points, at which genes are switched on/off, clearly determines the fate of what a cell does in terms of being functional or non-functional, due to disruption of that specific pathway. Recent research-work in this area (Bhojwani, 2015) provides strong evidence, toward identifying such components (associated with Wnt-signaling involved in Colorectal Cancer-CRC disease). These crucial elements indeed determined the genetic transformation of a “blank-slate” (“cells of origin” and/or putative “cancer stem cells”) or “primitive-state” epithelial cells to an intermediate adenoma/polyp (dyspastic), and later to a proliferative (hyperplastic) or cancerous (neoplastic) state. The idea is to re-iterate the power of genetics, in solving and filling the missing links of any developmental pathway involved in progression of a disease (in this case, CRC). A critical temporal requirement of certain molecules [Caesin-Kinase I (CKI) and Human-Discs-large (hDlg)] was finally established and these proteins were identified as “early” and “late” acting molecules respectively, in a very crucial developmental event, that basically transforms “polyps” to full-fledged “carcinomas” (epithelial cancers) in COLORECTAL tumors. The detection of these genetic and developmental parameters, served as a focal-point and a prominent diagnostic feature, for detection of effects, i.e., gain/loss of other components involved during progression of CRC disease. Coincidentally, the chromosomes on which these genes reside have been found to be dense and rich in SNPs (hot-spots), the details of which were published in a separate report (Patidar & Bhojwani, 2013). This work harnessed the potential of Genetics, Developmental Biology and Bio-Informatics tools to solve a long-standing puzzle in pin-pointing some genetic factors that were critically involved in the progression of CRC disease. The report has created enough impact, in terms of authentically suggesting, that it is only when we deploy a combinatorial approach towards certain complicated biological problems, can we successfully unveil the underlying mechanisms in greater details.

However, it is now conceived that, at the heart of every tumor lies a rare sub-population of cells (Cancer Stem Cells-CSCs), which give rise to most of the Cancers and are now the targets of investigation. Since no definitive markers or efficient labeling tools are available, this population of cells still remains elusive in both cancer and stem cell biology. Therefore, it would be critical to understand molecular differences between stem cells and cancer cells, which might be helpful in providing novel insights into the mechanism of tumorigenesis as well as potential therapeutic targets, in foreseeable future. We have come a long way in the stem cell advances over time. Very recent breakthroughs include: (a) The tuning and genetic re-programming of stem cells (iPS cells) by a handful of genetic factors (Takahashi et al., 2006, 2007; discussed in Bhojwani, 2008) and; (b) The transformation of cancerous cells to normal cells by reversing the genetic changes involved and also restricting the awry cancerous cells by using microRNAs (http://yournewswire.com/breakthrough-scientists-find-way-to-change-cancer-cells-into-healthy-cells/). My talk would shed light on how we could intelligently utilize these efficient tools together, to attack the “Bad seeds” in ways to cure the myriad diseases, like Cancer.

**Biography**

Dr. Jyoti Bhojwani, is presently a Faculty of Genetics/Bioinformatics/ Principal Investigator of the M.Tech Research Programs (Bio-Informatics) at University of Indore, India. She obtained her BSc (Bachelors degree) in Biological Sciences/Chemistry/Physics, MSc (Master’s degree) in Life-Sciences, and Doctoral degree (Ph.D.) at School of Life-Sciences, University Of Indore. She pursued her post-doctoral ventures at Max-Planck Institute for Biophysical Chemistry (FRG), University Of California-Irvine and University of Pittsburgh (USA). Currently, her projects mainly focus on translational-research and extrapolation of basic developmental mechanisms from model-systems like fruitfly (Drosophila) to human. Apart from this, her thrust areas of research interest include; Cancer Biology, Stem-Cell Biology and Homeotic-Gene Regulation. She is keen on studying in details the genetic factors, which presumably aid in understanding of mechanism by which “cancer stem cells” function in transforming a tissue from normal to cancerous states. Her research has a motive to further facilitate the perception of stem cell potential/mechanistic in areas of Regenerative Medicine, Translational Research and Anti-cancer therapy. Being involved in Clinical informatics, her students are also training a Cancer model and a Stem cell model, deploying Systems Biology approach and other Gene Networking BioInformatics tools. This novel area of research will hopefully lead to further understanding the tipping of balance from a stem cell/normal cell to a transformed cancer cell. Owing to her immense interest in science journalism and writing potential, she is now on the editorial board of five-six International Journals. Her Specialties Include: Research/Teaching/Mentoring/Science-Journalism.

### 3.7. Nanomedicine and Engineering

#### 3.7.1. ApoB-Peptide Conjugated Gold Nanoparticles Exhibit LDLR-Targeting in Malignant Glioma Cell and Transcytosis-Crossing in Blood Brain Barrier

Kim, J.-K. and Seo, S.J.

**Abstract**

ApoB peptide 29mer was LDLR binding element in natural low density lipoprotein. LDLR is abundant in BBB, and overexpressed in malignant brain glioma. ApoB-conjugated gold nanoparticles (ApoB@AuNP) were synthesized to develop nanobeacon in Coulomb nanoradiator (CNR) therapy for the treatment of malignant glioma. Transcytosis to BBB and cellular uptake were tested using TEER method and ICP-MS, respectively, and compared with bare gold nanoparticles. In order to present nanoradiator-mediated electron/X-ray photon emission, SH-PEG-hydrocynine 5.5 was conjugated to ApoB@AuNP. ROS-enhancement of hydrocyanine-conjugated ApoB@AuNP was measured in situ under traversing proton beam based on ROS-oxidant fluorescence of hydrocyanine.

BBB-transcytosis of ApoB@AuNP increased dose-dependently, demonstrated 3.4 fold augmentation compared to bare AuNP in 24 h post-incubation. Intracellular uptake in F98 glioma cell increased greatly by 10–20 fold compared to bare AuNP. CNR-mediated enhancement of ROS production was increased by 3.5 fold under 4-Gy traversing proton irradiation compared to non-treatment. In conclusion, ApoB@AuNP exhibited great potential as therapeutic nanobeacon with transcytosis-based BBB crossing and targeted delivery to malignant glioma for CNR therapy.

**Biography**

Professor Jong-Ki Kim is the chairman of Biomedical Engineering in School of Medicine, Catholic University of Daegu, based in Daegu City, South Korea. Professor Kim has published distinguished papers on the subject of Coulomb nanoradiator therapy and related physics. He is a recognized pioneer in the use of nanoradiator effects, giving many invited papers at international meetings around the world.After obtaining Ph.D. in Biophysics from the State University of New York at Buffalo in 1992, he spentpostdoctoral at Memorial Sloan-Kettering Cancer Center on NMR spectroscopy on DNA duplex containing photoadducts to reveal structural basis of DNA mutagenesis. He continued his research in MRI and MR spectroscopy at Catholic University of Daegu as Professor in School of Medicine since 1995. During last decade his research subject was extended to nanomedicine in the field of radiation impact on high-Z nanoparticles. Recently, he had spent visiting professor in Bioengineering, UC Berkeley. He pioneered in the development of site-specific Coulomb nanoradiator therapy using traversing Bragg-peak ion beam and targeted high-Z nanoparticles. Dr. Seo obtained Ph.D. in 2012 under guidance Prof Kim. Dr. Seo developed diffraction based synchrotron X-ray brain imaging and Coulomb nanoradiator therapy in a number of disease models including thrombosis and malignant gliom. He is currently a Research Professor in Biomedical Engineering at School of Medicine, Catholic University of Daegu.

#### 3.7.2. Hierarchical Micro/Nano-Porous Acupuncture Needles Offering Enhanced Therapeutic Properties

In, S.-I.

**Abstract**

Acupuncture has long been accepted as an effective therapy for the treatment of many functional disorders, such as pain and psychiatric disorders including anxiety and drug abuse. The invention of acupuncture as a therapeutic treatment is traced as far back as 6000 B.C., originating with the insertion of sharpened stones at specific acupuncture points. The ancient use of sharp stones as an acupuncture device is replaced by that of fine nee-dles made from various materials including bamboo, ceramic, bone, and plant thorns, with these in turn replaced by metal acupuncture needles, including those of gold, silver, copper, and stainless steel. The biological basis of acupuncture still remains unclear, however a considerable number of studies has established a general concept that acupuncture contributes to the neurochemical balance in the central nervous system (CNS) and recovery or maintenance of homeostasis via interactions between needles and the surrounding tissues. In our previous study we reported the activation of the A-beta afferent fiber (sensory nerve fiber) of the ulnar nerve promoting cellular activation by acupuncture at Shenmen (HT7) points for modulating cocaine-induced addictive behavior. Moreover, it is found that mechanoreceptors in the superficial and deep afferents of the ulnar nerve play a functional role in producing acupuncture effects during mechanical stimulation of HT7. Involvement of the afferent fibers in acupuncture is supported by studies investigating acupuncture-like stimulation of superficial or deep tissues for reducing micturition contraction of the urinary bladder and acupuncture analgesia abolished by blockade of afferents fibers from muscle. In acupuncture therapies manual manipulation of acupuncture needles is still the most practicable clinical procedure to enhance the stimulation intensity for improved therapeutic effects. Various needle parameters such as diameter depth of insertion number of needles used per session and needle surface modification have been investigated for improved acupuncture performance. These studies suggest that employing.

**Biography**

Professor SU-IL IN is the Dean of External and International Affairs at DGIST (Daegu Gyeongbuk Institute of Science and Technology) since 2016. He has been working at DGIST since 2012. He received his Ph.D. in Chemistry from the University of Cambridge in 2008. He then became a postdoctoral research associate at the Technical University of Denmark in 2010. He also joined the Department of Chemistry at Pennsylvania State University as a postdoctoral fellow before joining DGIST. Professor In’s current researches include synthesis and analysis of functional nano (bio)-materials for environmentally friendly renewable energy such as photovoltaic, heterogeneous catalysis and biocatalysts. A central goal of this work is relating surface structure/properties, size and composition to the catalytic activity and microbial fuel cell (MFC).

#### 3.7.3. Poly (Acrylic Acid)-Coated Iron Oxide Nanoparticles through Ligands Exchange for Biofilm Applications

Nie, L.

**Abstract**

Biofilms on cutaneous skin wounds and biomaterials implants are hard to eradicate with antibiotics due to antibiotic resistance and the lack of new antibacterial agents. Such biofilms could be broken by using the nanotechnology, without resorting to the antibiotics, such as metal-based nanoparticles. Poly (acrylic acid) (PAA) functionalized iron oxide nanoparticles were synthesized through a two-step process in this paper. Firstly, Oleic acid-coated nanoparticles were synthesized via a thermal decomposition of iron oleate, then, through ligands exchange, PAA-coated iron oxide nanoparticles were synthesized with using THF as solvent. Finally, the toxicity of PAA-coated iron oxide nanoparticles on skin cells and efficacy of nanoparticles against bacteria were evaluated. The results showed that the HaCat cell had a good viability while culturing with nanoparticles for 3 days, moreover, the PAA-coated iron nanoparticles caused bacteria killing. The PAA-coated iron oxide nanoparticles we prepared might be the ideal antibacterial treatment, had a better activity than iron oxide nanoparticles alone.

**Biography**

Dr. Lei Nie obtained the Ph.D. in nanotechnology and Science from Huazhong University of Science and Technology in 2013, after that, he started work as an assistant professor in Ningbo Institute of Materials Technology & Engineering, Chinese academy of science, during his time in Ningbo, he continued his research in tissue engineering, growth factors delivery system, and biodegradable copolymer functionalization. In 2015 he moved to Free University of Berlin in Berlin, Germany where he focused his research on nanoparticles preparation and functionalization for biofilms application, and distinguish the influences of hydrophilic surface functionalization and transfer in aqueous media on the magnetic properties and the oxidation state of iron oxide nanoparticles. He is currently an associate professor in Xinyang Normal University at China, he focuses his researches on polymer synthesis for multi-function hydrogels, nanoparticles functionalization for drug delivery, biodegradable scaffold for 3D cell culture and tissue engineering.

### 3.8. Physical Therapy and Rehabilitation

#### 3.8.1. Evaluating and Treating Shoulder Dysfunction through a Regional Interdependence Approach

Cramer, J.

**Abstract**

Traditional orthopedic evaluations often lead to inaccurate diagnoses of musculoskeletal injuries as clinicians tend to gear their evaluation and treatment plan towards the local symptomatic area. Applying this process may generate misdiagnoses or involve prolonged unnecessary treatments away from the actual problem. These concerns especially arise with the intricacies of the shoulder complex. Glenohumeral (GH) range of motion (ROM) asymmetries associated with total rotational motion (TROM) deficits in overhead athletes demonstrated increases in injury rates. TROM is the unilateral sum of GH internal/external rotation. TROM deficit occurs when there is an asymmetrical difference greater than 5°. Pathologic GIRD (pGIRD) occurs when IR in the dominant shoulder is greater than 18–20° and a bilateral TROM asymmetry greater than 5°. Common treatment interventions consist of static stretching focused on increasing IR and typically range from four to six weeks. Regional interdependence (RI) is a concept explaining how seemingly unrelated impairments may be directly or indirectly related to the patient’s primary complaint. Utilizing this concept, Total Motion Release (TMR^®^) is an evaluation and treatment intervention designed to restore musculoskeletal asymmetries by correcting restricted ROM, speed and stability of movement, and sensations such as pain or tightness in a quicker hands-free manner.

**Biography**

Joshua Cramer is the head athletic trainer at Germantown Academy in Fort Washington, Pa. He also serves as the head athletic trainer for the Philadelphia Freedoms. Joshua attended Catawba College in North Carolina where he graduated with degrees in athletic training and communications and a minor in speech in 2005. He continued his education at the University of Florida receiving his masters of Science degree in applied physiology and kinesiology with an emphasis on athletic training. During this time, Joshua had the pleasure of working with the baseball men’s basketball teams in an athletic training and strength and conditioning capacity respectively. He has since spent the past three plus years working towards his doctorate in athletic training where he completed a clinical residency and is currently finishing his dissertation. Joshua has worked at various sports levels including public and private secondary schools, divisions I and II collegiate sports and professional sports. His areas of expertise surround the shoulder, concussions and manual therapy. He holds many certifications including athletic training, exercise physiology, strength and conditioning, orthopedic and orthopedic brace technology, corrective exercise, sports nutrition as well as is an experienced manual therapist utilizing techniques like: Myofascial Release, Primal Reflex Release Technique, Applied Movement Neurology, Mechanical Diagnosis & Therapy, Positional Release Technique, Neurodynamics, Mulligan Concept, Myokinesthetics, etc…

#### 3.8.2. Differentiation of Neural Reflex and Muscular Intrinsic Contributions to Rigidity in Parkinson’s Disease

Xia, R.

**Abstract**

Parkinson’s disease (PD) is a progressive neurodegenerative disease characterized by rigidity, bradykinesia, resting tremor, and postural instability. Rigidity, defined as an increased resistance to passive movement of a joint, progresses faster than other motor signs in PD. Rigidity is attributable to both exaggerated neural reflex and altered muscle mechanical properties. However, little is known about the contributions of individual components to rigidity. Further, there is no evidence regarding the effects of dopaminergic medication on individual components. Objectives of this study were to quantify the contributions of neural reflexes and intrinsic muscle properties to rigidity, and investigate the effects of medication on each contributing component. Joint torque and muscle activities of the wrist in 14 patients and 14 controls were measured during externally-induced movements. Each subject with PD was tested in Off- and On-medication states. A system identification technique was applied to differentiate and quantify the neural reflex and intrinsic mechanical components. A mixed model of analysis of variance (ANOVA) was performed to compare the differences between the two components of rigidity for both groups, and to compare between the Off- and On-medication states for patients. The results showed that reflex and intrinsic components are comparable (*p* > 0.05), and both are enhanced in subjects with PD than in the controls (*p* < 0.05). Medication decreased the reflex component of rigidity (*p* < 0.01). It is concluded that both reflex and intrinsic factors are responsible for rigidity. Present findings are clinically significant as they may provide guidance in development of effective therapeutic interventions. Further, this line of research is currently in progress by examining effectiveness of physical therapy programs (such as muscle stretching and therapeutic taping) on reducing neural reflex and intrinsic components in parkinsonian rigidity.

**Biography**

Dr. RuiPing Xia received her bachelor’s and master’s degrees in Biomedical Engineering from Tianjin University, China, and her doctoral degree (Ph.D.) in Neurophysiology from the University of Bristol, UK. Upon completing her doctoral study, Dr. Xia worked as a Research Scientist at the Institute of Neurology, University College London, UK. She also completed a post-doctoral research fellowship in neurological rehabilitation at Northwestern University’s Feinberg School of Medicine and the Rehabilitation Institute of Chicago, USA. Dr. Xia has been a core faculty in Physical Therapy since 2003. Her current and previous teaching activities in physical therapy curriculum include motor control and motor learning, neuroscience, critical inquiry and evidence-based practice, and expertise practice in physical therapy. In addition to teaching, Dr. Xia has mentored doctoral physical therapy students, research associates, and post-doctoral fellows through their research projects. Dr. Xia’s research interests focus on motor control, sensorimotor integration, neural rehabilitation, and Parkinson’s disease. Her research has generated numerous peer-reviewed articles published in prestigious journals such as Clinical Neurophysiology, Experimental Brain Research, Journal of Physiology (London), and Physical Therapy journal. Her publications are widely cited by investigators worldwide. She has also served as the principal investigator of several funded projects—including a few sponsored by the National Institutes of Health (NIH), USA. She is the recipient of several Research Awards and Fellowships.

#### 3.8.3. Quantumphysical Efficacy of Homeopathy by Magnetic Photons

Lenger, K.

**Abstract**

The nature of homeopathy using dilutions beyond the Avogadro number is clarified since Lenger’s detection of magnetic photons in the MHz-region of homeopathic remedies by two different magnetic resonance methods: by the Tesla-flatcoil system and delayed luminescence (DL) using a modified photomultiplier. Separation of the newly detected magnetic photons from their carrier substance ethanol or saccharose globules took place when the measuring system has a bigger resonating frequency field than the field between carrier substance and photons. Characteristic frequency spectra and the potency levels were measurable by the number of photons, taking DL or by the characteristic size of the magnetic frequency field separating the photons in dependence on the potency level and on the potentized substance. Six unknown remedies could be identified by DL. The Law of Similars (Hahnemann 1796) can be expressed like this: the frequencies of the patient must match the frequencies of the remedies to get in resonance: either to attenuate the ill making frequencies or to enhance the amplitude of both. Since Einsteina human body is an electromagnetic wave package. It is assumed that healing takes place firstly quantumphysically on the energy level; later on the pathological pathways are regulated. This is in agreement with Popp’s quantumphysical theory about health and illness and Förster saying that each chemical reaction takes place on a higher energetic level of the atoms. The uptake of photons is necessary in biological systems to keep them alive in steady-state.

Because of its efficacy according to the resonance principle homeopathy is a regulation therapy curing both: hyperfunction and hypofunction of a pathological pathway and consciousness applying additional potentized substrates, inhibitors and enzymes: Hashimoto disease supported by the substrates of the thyroid-pathway, asthma was healed by using the highly potentized substrates of the respiration chain and those of the glycolysis, paralyses require mostly the neurotransmitter Acetylcholine which biosynthesis is supported by highly potentized substrates and inhibitors of glycolysis and fatty acid oxidation. The psychological problems of the patients are simultaneously healed by the applied remedies. Homeopathic healing means that body and consciousness are treated as one because homeopathy is a holistic medicine.

**Biography**

Karin Lenger studied Biochemistry at the universities of Tübingen and Cologne/Germany. She worked as a Scientific Assistant at the Medical University of Lübeck/Germany for 12 years performing her biochemical enzymatic studies: enzymatic gene regulation, cancer research, enzymatic mechanisms of steroid hormones. She started her homeopathic career as a Lecturer for classical homeopathy at DHU (Deutsche Homöopathie Union = German Homeopathy Union) in Karlsruhe 1986–1994. Since 1995, Dr. Lenger has been working as a practising doctor and Lecturer for classical homeopathy in Europe. Over the years she has developed the “biochemical homeopathy” by using substrates of pathological enzymes in high levels of potentization. She detected photons in high homeopathic potencies by scientific proof, by two magnetic resonance methods. She has developed a model of physical and biochemical function of homeopathy. She has a lot of publications and had been invited for speeches on many world congresses in Europe, USA, Shanghai, Dubai, Hongkong, Nangjing.

#### 3.8.4. A Review of the Current Status of Bamboo Usage with Special Emphasis on Orthopedic Rehabilitation

Reed, K.S.

**Abstract**

The number of people with gait dysfunction is fast growing due to population growth, war, ageing and accidents. The cost of orthopedic devices essential to restore function and improve quality of life is not affordable for many. Earlier research in this area suggests that bamboo is a suitable material for orthopedic appliances, especially exoskeleton. In order to further advance research in this area, the role of bamboo as a structural material, with special emphasis on orthopedic appliances was carried out in this work. It was found that bamboo is being extensively used as infrastructure material in Asia and Africa due to its excellent mechanical properties. Its usage has ranged from simple fence construction to bridges. The use of bamboo as an orthopedic rehabilitation aid is prevalent in developing world where traditional bonesetters have utilized bamboo as a method of splinting for fracture treatment and management. Despite the great progress made in the usage of bamboo as infrastructure materials, the application in orthopedic rehabilitation appliances is limited to traditional practices due to a lack of standardization. Our work revealed that bamboo can be used in a variety of ways for orthopedic purposes, and can be classified into three categories based on the complexity of design and manufacturing.

**Biography**

Kischa S. Reed, PT, DPT, COMT is an Assistant Professor in the Division of Physical Therapy since 2012. Dr. Reed received her B.S degree in Physical Therapy from the Florida Agricultural and Mechanical University in 1998 and earned her transitional Doctor of Physical Therapy degree from Utica College in 2011. As a Certified Orthopedic Manual Therapist (COMT), Dr. Reed is able to combine the latest Evidence-Based research in Manual Therapy/Physiotherapy with the Maitland Approach of assessment and treatment which emphasizes clinical decision-making, advanced orthopedic clinical practice, accurate techniques of assessment/treatment, effective treatment progression and safety, to provide advanced treatment techniques in acute, chronic, and post-surgical spine rehabilitation. Having this knowledge of manual skills and core competencies align well with her current teaching responsibilities in Analysis of Human Motion (Biomechanics/Kinesiology), Orthopedic Physical Therapy, Vertebral Manipulation and Mobilization, and Physical Agents in the physical therapy program. Dr. Reed is a member of the Florida Physical Therapy Association (FPTA), where she formerly served as a Florida Assembly representative.

#### 3.8.5. Understanding Human Postural Sway in Health and Disease

Gurses, S.

**Abstract**

Human postural sway demonstrates complex dynamical characteristics where postural control system utilizes the *perceived-self* through a redundant motor system by fusing senses relaying different originated information in a variety of domains; such as time, frequency, and symbolic spaces. The complex dynamics have been investigated by constructing its phase-space representation from Antero-Posterior Center-of-Pressure (*CoP_x_*) trajectories, where a characteristic pattern has been identified depending on nonlinear dynamics (slightly positive largest Lyapunov exponent causing deterministic chaos), e.g., caused by sensory thresholds demonstrating individual signature properties. Furthermore, correlation dimension estimates computed from *CoP_x_* trajectories have been shown to reveal that human quiet standing demonstrates multiple degrees of freedom dynamics having a fractal structure with a considerable level of noise embedded in the signal (with individual characteristics). On the contrary, Bilateral Vestibular Loss patients’ (BVL) *CoP_x_* trajectories presented comparatively poor dynamics having lost some characteristic low frequency-bands in their spectral analyses against some speedy frequency-bands gained (treated as compensatory/*adapted ill-postural* behavior), whose unaltered presence has been proposed (through thermodynamics based nonlinear metrics) to work as a potential for ecological source of adaptive information.

**Biography**

Dr. Senih Gürses having graduated from Medical College of Hacettepe University, Ankara in 1986, worked as a physician for about 10 years while completing his MSc Thesis (Infra-red Mapping of Cerebral Audio-Cortex of a Rabbit) in Biomedical Engineering at Bosphorus University, İstanbul and the University of Tokyo. He completed his Ph.D. Thesis on Postural Dynamics and Stability at Middle East Technical University, Ankara in 2002 and moved to Rehabilitation Institute of Chicago as a post-Doctoral Researcher. There he studied nonlinear dynamical characteristics of human postural control, especially sensory-motor performance investigated in healthy versus vestibular loss subjects and cerebellar patients. In 2005, he returned to Middle East Technical University as an Assistant Professor and lectured in Principles of Mechanics and Biomechanics. From there on, a postural research team established by Dr. Gürses collaborating with Oto-Rhino-Laryngology and Neurology Departments of Gülhane Training and Research Hospital and Physical Medicine and Rehabilitation Department of Gazi University Medical College studied different aspect of sensory fusion and multi-integration, perception/action in postural control (vestibular-somatosensory interaction, foot somatosensory in postural control) in healthy and diseased conditions especially in view of ecological adaptation revealed through thermodynamically based nonlinear dynamical metrics utilized by information (ergodic) theory.

## 4. Author Affiliations

- Chen, J.-H., National Yang-Ming University, Taiwan
- Cho, K. Yonsei University, Korea
- Litscher, G. President of ISLA (International Society of Medical Laser Applications), Medical University of Graz, Austria
- Caesar, I. Matrix City LLC, Russia
- Mei, L. Beth Israel Deaconess Medical Center, USA
- Wen, G. South China University of Technology, China
- Segura-Saldana, P. Universidad Peruana Cayetano Heredia, Perú
- Xu, S. Peking University, China
- Lee, C.-J. Fu-Jen Catholic University, Taiwan
- Heer, R. Austrian Institute of Technology, Austria
- Simone, G. Northwestern Polytechnical University, USA
- Singh, A. Harvard University, USA
- Stanciu, G.A. University Politehnica of Bucharest, Romania
- Song, W. Shenzhen University, China
- Sun, R. Chairman & CEO, Xi’an Pillar Bioscience Co. Ltd., China
- Pinchuck, L. President & CEO, Innovia, LLC
- López-Bayghen, E. Cinvestav-IPN, México
- Cedillo, L. Cinvestav-IPN, México
- Ocampo, A. Cinvestav-IPN, México
- Camargo, F. Cinvestav-IPN, México
- Frighetto, L. Hospital Moinhos de Vento, Brazil
- Weinshilboum, R.M. Mayo Clinic College of Medicine, USA
- Wang, L. Mayo Clinic, USA
- Bou-Saïd, B. LaMCoS-INSA Lyon, France
- Milia, P. Italy University of Perugia, Italy
- Lee, Y.-H. National Central University, Taiwan
- Wang, S. Northwestern Polytechnical University, China
- Teng, J. Louisiana State University Health Sciences Center in Shreveport, USA
- Herrera, G. Louisiana State University Health Sciences Center, USA
- Turbat-Herrera, E. Louisiana State University Health Sciences Center, USA
- Chen, H.H. Founder and CSO, StemEasy Biotech Ltd., China
- Purwati Stem Cell Research and Development Center, Universitas Airlangga, Indonesia
- Yang, Y. The University of Hong Kong, Hong Kong
- Li, J. Zhengzhou University, China
- Nordon, R.E. University of New South Wales, Australia
- Chen, H. The Affiliated Drum Tower Hospital of Nanjing University Medical School, China
- Wang, H. East China Normal University, China
- Huang, W.-X. Tsinghua University, China
- Shan, X. Shandong Normal University, China
- Wu, Z. Canterbury Pharmaceuticals, New Zealand
- Zheng, W. University of Macau, China
- Xie, L. Molecular Medicine of University of Georgia, Georgia
- Bhojwani, J. Indore University, India
- Kim, J.-K. Biomedical Engineering and Radiology, School of Medicine, Catholic University of Daegu, Korea
- Seo, S.J. Biomedical Engineering and Radiology, School of Medicine, Catholic University of Daegu, Korea
- In, S.-I. DGIST, Korea
- Nie, L. Xinyang Normal University, China
- Cramer, J. Germantown Academy/Philadelphia Freedoms, USA
- Xia, R. University of Saint Mary, USA
- Lenger, K. Institute for Scientific Homeopathy, Germany
- Reed, K.S. Florida Agricultural and Mechanical University, USA
- Gurses, S. Middle East Technical University, Turkey

